# Energy-efficient intrusion detection with a protocol-aware transformer–spiking hybrid model

**DOI:** 10.1038/s41598-026-37367-4

**Published:** 2026-02-03

**Authors:** M. Ganesh Karthik, Vijay Keerthika, Srihari Varma Mantena, D. Siri, Lakshmi Prasanna Yeluri, Kranthi Kumar Lella, B. Rama Ganesh

**Affiliations:** 1 GITAM School of Computer Science and Engineering, GITAM University-Bengaluru Campus, Bengaluru, India; 2 Department of CSE-AIML, MLR Institute of Technology, Hyderabad, India; 3 Department of Computer Science and Engineering, SRKR Engineering College, Bhimavaram, 534204 India; 4 Department of CSE, Gokaraju Rangaraju Institute of Engineering and Technology, Hyderabad, India; 5 Department of Computer Science and Information Technology, Koneru Lakshmaiah Education Foundation, Hyderabad, 500043 India; 6 Manipal Institute of Technology, Manipal Academy of Higher Education, Manipal, 576104 India; 7 Department of Computer Science and Engineering, Sri Venkatesa Perumal College of Engineering & Technology, Puttur, Andhra Pradesh 517583 India

**Keywords:** Intrusion detection, Transformer, Spiking neural networks, Protocol-aware normalization, Energy efficiency, Engineering, Mathematics and computing

## Abstract

Recent intrusion detection studies have achieved high accuracy using deep learning and transformer-based models; however, many approaches suffer from high computational cost, limited energy efficiency, and poor detection of rare attack classes in imbalanced network traffic. To address these challenges, this study proposes a Transformer-Augmented Spiking Neural Network (TASNN) that integrates attention-driven contextual modeling with energy-efficient spiking computation for intrusion detection systems (IDS). The framework incorporates Protocol-Aware Adaptive Normalization (PAAN) and Pseudo-Flow Reconstruction (PFR) to improve robustness to heterogeneous traffic patterns. An adaptive spike encoding strategy, including Multi-Scale Adaptive Spike Encoding (MASE) and Eventified Delta Coding (EDC), converts tabular features into sparse spiking representations. In addition, a Cross-Modal Gating (XMG) mechanism dynamically regulates spiking activity, while Spike-Aware Information Fusion (SAIF) supports stable and interpretable feature selection. Experimental evaluation on benchmark datasets demonstrates that TASNN achieves improved classification performance and reduced computational overhead compared to existing methods, highlighting its suitability for energy-constrained and edge-based intrusion detection scenarios.

## Introduction

With the rapid proliferation of Internet-connected devices, networks are becoming increasingly complex and thus more vulnerable to diverse forms of cyberattacks^1^. Traditional intrusion detection systems (IDS) that rely on signature-based mechanisms are no longer sufficient, as modern threats are growing more sophisticated and continuously evolving. This creates an urgent need for smarter, adaptive, and more reliable solutions^2^. Over the past two decades, IDS research has progressively incorporated learning-based methods that adapt to dynamic traffic patterns and support decision-making under multiple constraints. Despite these advances, several critical challenges remain unresolved, including energy efficiency, explainability, class imbalance, and robustness under noisy or adversarial environments^3^. These challenges underscore the necessity of developing hybrid solutions that integrate the strengths of different paradigms while overcoming the limitations of conventional approaches^4^.

Early intrusion detection approaches employed machine learning algorithms such as K-Nearest Neighbours (k-NN), Decision Trees, Support Vector Machines (SVMs), and Random Forests (RF). However, these models often struggled with scalability and exhibited poor performance on imbalanced attack classes such as Remote-to-Local (R2L) and User-to-Root (U2R)^5^. To address these shortcomings, researchers turned to deep learning methods, including autoencoders, LSTMs, CNNs, and RNNs. These approaches improved the detection of frequent attack types, such as Denial-of-Service (DoS) and Probe attacks^6^, but at the cost of high computational demands, limited interpretability, and poor recognition of rare or sophisticated intrusions.

Recently, transformer-based models have been applied to IDS tasks due to their strong ability to capture sequential and contextual information. Their self-attention mechanism excels at identifying long-range dependencies and extracting salient features^7^. Studies have shown that transformers outperform CNNs and RNNs in terms of accuracy and interpretability for intrusion detection. However, their resource-intensive nature raises concerns about their suitability for neuromorphic or edge-computing environments such as IoT networks^8^.

In parallel, spiking neural networks (SNNs) have gained attention for their event-driven and energy-efficient computation, which mimics biological neuronal processing. Unlike conventional ANNs, SNNs operate on discrete spike events over time, enabling sparse and asynchronous computation^9^. Neuromorphic hardware platforms such as Intel Loihi and SpiNNaker leverage SNNs to achieve real-time detection with minimal power consumption. Beyond efficiency, the temporal dynamics of SNNs also enable effective representation of bursty or irregular traffic patterns^10^. Nevertheless, standalone SNNs face challenges when applied to tabular datasets like NSL-KDD, as they struggle to capture high-level semantic relationships and require additional encoding schemes for categorical data.

To address these limitations, several studies have explored hybrid frameworks that combine the contextual learning capacity of transformers with the temporal efficiency of SNNs^11^. While such models demonstrate promise for anomaly detection, challenges remain in designing explainable feature selection methods, robust spike encoding strategies, and effective attention mechanisms that integrate seamlessly with spiking dynamics^12^. Moreover, issues such as protocol-specific bias, feature imbalance, and incomplete temporal flow reconstruction persist. For example, training data often overrepresents TCP records, whereas ICMP and UDP traffic is underrepresented^13^. Similarly, rare attack classes such as R2L and U2R remain difficult to detect, necessitating robust feature engineering, noise-resilient normalization, and protocol-aware resampling techniques.

Conventional models such as Random Forests and Gradient Boosted Trees also encounter difficulties in capturing high-dimensional feature interactions and generalizing across datasets^14^. Hybrid CNN-LSTM methods improve feature extraction but demand significant computational resources. Transformer-based IDS frameworks achieve high scalability and accuracy but lack energy efficiency, interpretability, and real-time responsiveness. Likewise, SNN-based intrusion detection models exploit event-driven encodings to reduce energy use^15^, yet they often yield poor accuracy on high-dimensional tabular data. Their adoption in practice is limited by the absence of attention-guided feature refinement and efficient spike-encoding strategies^16^.

Despite notable advances in intrusion detection research, several key problems remain insufficiently addressed by existing approaches:

- Limited detection reliability for minority attack classes such as R2L and U2R due to severe class imbalance.
- Protocol-agnostic preprocessing that introduces feature bias across heterogeneous network traffic.
- High computational and energy costs of deep and transformer-based IDS models, restricting real-time and edge deployment.
- Insufficient modeling of short-range temporal interactions in tabular intrusion datasets.
- Unstable feature relevance and poorly calibrated decision scores affecting threshold-based evaluation.

These unresolved issues motivate the need for improved intrusion detection frameworks that balance accuracy, robustness, and efficiency.

It is therefore evident that a new hybrid model is required—one that combines the interpretability and contextual strength of transformers with the efficiency and temporal modelling power of SNNs^17^. The proposed framework introduces several novel components to overcome existing challenges. Protocol-Aware Adaptive Normalization (PAAN) ensures fair normalization across protocol strata, mitigating biased spike encodings. Pseudo-Flow Reconstruction (PFR) enhances temporal context by grouping records into micro-flows. Eventified Delta Coding (EDC) and Multi-Scale Adaptive Spike Encoding (MASE) convert tabular data into efficient spike trains. A FlowToken Transformer Encoder (FTE) improves contextual embeddings, while Cross-Modal Gating (XMG) dynamically regulates spike activity based on transformer attention. Finally, Stable-PAAN Subset (SPS) and Spike-Aware Information Fusion (SAIF) stabilize feature selection and significantly enhance detection of rare attack classes.

The contributions of the Transformer-Augmented Spiking Neural Network (TASNN) can be summarized as follows:

- Energy-aware hybridization – combining transformer attention with event-driven spiking neurons to balance accuracy and efficiency.
- Protocol-sensitive preprocessing – employing PAAN and PRE for robust normalization and categorical feature embedding.
- Temporal flow reconstruction – leveraging PFR to capture pseudo-flow contexts and rare attack patterns.
- Efficient spike encoding – using MASE and EDC to generate compact and informative spike trains.
- Explainable feature selection – integrating SAIF and SPS for stable, attention-driven feature refinement.
- Robust generalization – demonstrating superior performance across NSL-KDD, KDDTest + 21, and CICIDS datasets, with resilience to noise, imbalance, and adversarial perturbations.

The remainder of this article is structured as follows. “Related work” reviews related work on ML-, DL, and SNN-based intrusion detection methods. “Proposed model” details the proposed TASNN methodology, including preprocessing, spike encoding, hybrid design, and feature selection. “Results and discussion” presents experimental results, including classification metrics, ROC/DET curves, attention visualizations, and neuromorphic energy analysis. “Discussion over generalizability” discusses adversarial robustness, cross-dataset generalization, and stability. Finally, “Limitations” concludes.

## Related work

This section reviews prior research on intrusion detection systems (IDS) with the objective of identifying unresolved challenges in existing approaches rather than merely summarizing reported performance. The discussion focuses on machine learning, deep learning, attention-based, and hybrid IDS models, emphasizing their limitations with respect to class imbalance, protocol heterogeneity, computational efficiency, temporal modeling, and reliability of decision scores. Works are analyzed in the context of intrusion detection, and methodological gaps are explicitly highlighted to motivate the proposed approach.

An improved Long Short-Term Memory (LSTM) system for detecting anomalous network activity was proposed by Dash et al.^18^. The model optimizes LSTM hyperparameters using three different optimization methods: Particle Swarm Optimization (PSO), JAYA, and Salp Swarm Algorithm (SSA). The datasets employed in this study include NSL-KDD, CICIDS, and BoT-IoT. Accuracy, Precision, Recall, F-score, and Receiver Operating Characteristic (ROC) are the performance metrics used to evaluate the model. The PSO-LSTMIDS, JAYA-LSTMIDS, and SSA-LSTMIDS approaches were compared, and simulation results showed that SSA-LSTMIDS outperformed the others across all datasets.

Hui and Chiew^19^ examined several traditional machine learning techniques—such as Decision Trees, Naive Bayes Trees, Random Forests, Random Trees, MLP, and Support Vector Machines—for their effectiveness in binary and multi-class intrusion detection. They further proposed a CNN-LSTM-SA deep learning approach, which consistently achieved superior detection results compared to traditional methods. By integrating self-attention (SA) with CNN and LSTM, the proposed model extracts more correlated and discriminative features. Using the NSL-KDD dataset as a baseline, the findings indicate that the CNN-LSTM-SA approach could significantly improve the performance of network intrusion detection systems (NIDSs).

Cai et al.^20^ introduced a novel method to represent network traffic features in an enhanced RGB image format by combining global and local information. During the data augmentation phase, they adopted a denoising diffusion probabilistic model (DDPM) instead of conventional augmentation techniques such as VAE or GAN. By incorporating learnable variance parameter strategies and cosine noise addition, the DDPM generates higher-quality images. Their dual-attention residual network (DDP-DAR) enhances intrusion traffic detection accuracy by leveraging a multilayer network with a dual-attention mechanism. Extensive experimental results demonstrate that DDP-DAR outperforms state-of-the-art data augmentation–based approaches in terms of Accuracy, F1-measure, False Positive Rate (FPR), and ROC-AUC, while also delivering more consistent detection results.

Tahir et al.^21^ proposed Deep Learning-Based Missing Data Imputation (DMDI), a new approach for improving input data quality by efficiently handling missing values. To enable thorough testing and comparison, they applied the Random Missing Value (RMV) algorithm to simulate missing data. DMDI integrates Gradient Boosting and a stacked denoising autoencoder to enhance imputation accuracy. The method was evaluated using the NSL-KDD and UNSW-NB15 datasets, where five classifiers—SVM, KNN, Logistic Regression, Decision Tree, and Random Forest—achieved significantly higher performance after DMDI-based imputation. Accuracy improved from an average of 0.95 to 0.97 across classifiers. Extensive testing in Python 3 confirmed that the model was more resilient and effective. This highlights the importance of accurate data imputation for deep learning–based anomaly detection and provides a robust technique for IDS datasets.

Manivannan and Senthilkumar^22^ developed the ARN-FOX approach, an adaptive recurrent neural network optimized using the fox algorithm. The ARNN-FOX system aims to enhance network security by efficiently identifying and classifying intrusions. Data normalization is applied to preprocess the input, and feature extraction is performed using the GLCM technique. The ARNN’s hyperparameters are fine-tuned using the FOX optimizer. Benchmark datasets were employed to evaluate performance, and results show that ARNN-FOX achieves superior metrics across recall, precision, F1-score, sensitivity, and specificity. Compared with RNN, CNN-LSTM, DASO-RNN, and ChCSO-LSTM, the proposed ARNN-FOX model yielded accuracy improvements of 15.12%, 8.79%, 6.45%, and 4.21%, respectively. In terms of specificity, it surpassed RNN by 32.43%, CNN-LSTM by 8.89%, DASO-RNN by 3.16%, and ChCSO-LSTM by 2.08%, establishing its effectiveness for network intrusion detection.

To further improve detection by leveraging structural information in data flows, Deng and Huang^23^. proposed the Edge-featured Multi-hop Attention Graph Neural Network for Intrusion Detection System (EMA-IDS). By incorporating node and edge properties and enhancing computation through attention propagation, EMA-IDS effectively exploits data characteristics. Experiments conducted on NF-CSE-CIC-IDS2018-v2, NF-UNSW-NB15-v2, NF-BoT-IoT, and NF-ToN-IoT datasets showed that EMA-IDS outperformed existing models.

Kharoubi et al.^24^ designed a CNN-based deep learning architecture for NIDS, tailored to IoT environments. The model demonstrated high accuracy, flexibility, and reduced latency in both binary and multi-class classification tasks when tested on CICIoT2023, Edge-IIoTset, and CICIoMT2024 datasets. Experimental results confirmed that the proposed CNN-based NIDS consistently surpassed state-of-the-art methods across accuracy, precision, recall, and F1-score, providing an effective real-time IoT security solution.

Ahmed et al.^25^ evaluated two state-of-the-art ensemble classifiers—eXtreme Gradient Boosting (XGBoost) and Light Gradient Boosting Machine (LGBM)—for detecting a wide range of cyberattacks. Their study revealed critical vulnerabilities in IoT traffic and demonstrated superior performance of both classifiers, with average accuracies of 99.553% and 99.651%, respectively. Results showed reduced false positives and false negatives, making them highly suitable for securing IoT networks. The study also presented comprehensive analyses of class balance and feature importance using the RT-IoT2022 dataset and applied SMOTE to mitigate imbalance issues. Their findings highlight the potential of ensemble models in building AI-powered defense systems against evolving cybersecurity threats.

Finally, Thaljaoui^26^. proposed a hybrid CNN-LSTM model with Bayesian optimization for hyperparameter tuning, aimed at improving IoT threat detection. The model was trained and tested on the UNSW-NB15 benchmark dataset and evaluated using multiple metrics, including F1-score, recall, accuracy, and precision. Comparative analysis demonstrated that the proposed approach outperformed other models, confirming its effectiveness in intrusion detection and its ability to accurately identify diverse types of cyberattacks.

Despite significant progress, the reviewed intrusion detection studies exhibit several common limitations. Traditional machine learning and early deep learning models report degraded detection performance for minority attack classes such as R2L and U2R due to severe class imbalance and limited feature discrimination. CNN- and LSTM-based IDS frameworks improve representation learning but incur high computational cost and limited scalability in real-time environments.

Recent transformer-based IDS models enhance contextual learning but introduce substantial computational overhead and lack energy-aware design, restricting their applicability in resource-constrained settings. Spiking neural network–based approaches emphasize low-power computation; however, they struggle with high-dimensional tabular intrusion data and lack attention-guided feature refinement. Furthermore, most existing studies do not explicitly address protocol-aware preprocessing or calibrated probabilistic outputs, leading to unreliable threshold-based evaluation and inconsistent ROC behavior.

Based on the above analysis, a clear research gap emerges. Existing IDS solutions do not jointly address protocol-aware feature handling, reliable detection of minority attack classes, energy-efficient computation, and calibrated decision-making within a unified framework. This gap motivates the development of a hybrid intrusion detection model that integrates contextual learning, temporal efficiency, and robust probabilistic evaluation while remaining suitable for real-time and resource-constrained environments.

## Proposed model

**Fig. 1:**
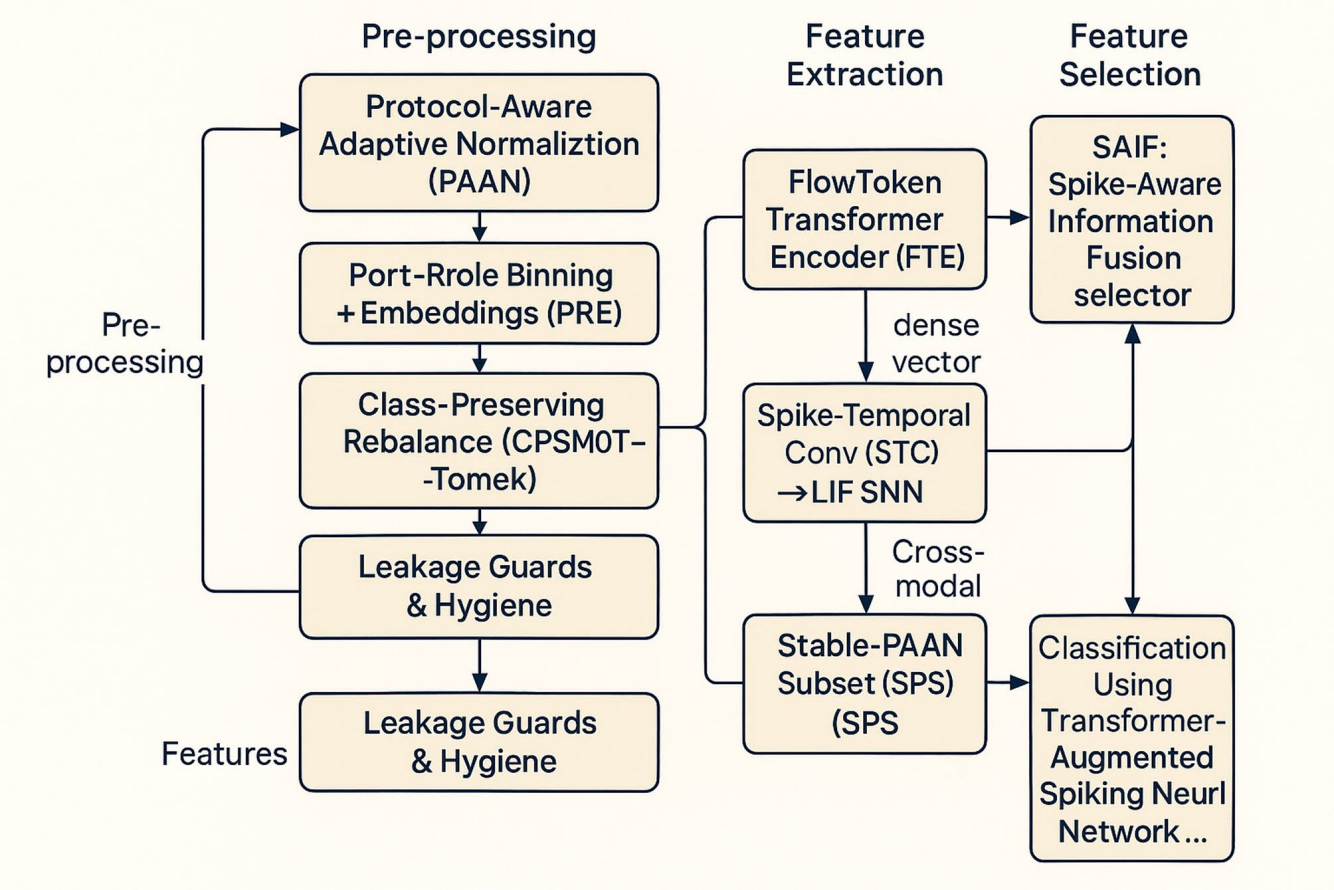
Workflow of the proposed model.

This section presents a complete, end-to-end framework that marries protocol-aware tabular modelling with energy-efficient spiking computation. The pipeline is purpose-built for NSL-KDD, where records are packet/connection summaries with rich categorical fields and heterogeneous continuous ranges. Our design injects temporal and protocol semantics *before* learning (so the model “sees” what matters), encodes features into sparse spike events (to exploit SNN advantages), and binds a compact Transformer to an LIF SNN through cross-modal gating (to focus spiking on suspicious interactions) and it is shown in Fig. 1.

Novelty arises from: Protocol-Aware Adaptive Normalization (PAAN), Port–Role Embeddings (PRE), Class-Preserving SMOTE with Tomek cleaning (CPSMOTE-Tomek), Pseudo-Flow Reconstruction (PFR), Multi-Scale Adaptive Spike Encoding (MASE) with Eventified Delta Coding (EDC), the FlowToken Transformer Encoder (FTE), Spike-Temporal Conv (STC) → LIF SNN, Cross-Modal Gating (XMG), and Spike-Aware Information Fusion (SAIF) feature selection with Stable-PAAN Subset (SPS) consolidation.

### Notation and problem setting

Let $\:{D=\left\{\right({x}_{i},{y}_{i}\left)\right\}}_{i=1}^{N}$​ be NSL-KDD samples with labels $\:{y}_{i}\in\:\left\{\mathrm{0,1}\right\}$ (normal/anomaly) or multi-class attack family. Decompose $\:{x}_{i}=[{x}_{i}^{cont},{x}_{i}^{cat}]$ into continuous and categorical subsets. Let $\:p\triangleq\:\:number$ of raw features; after preprocessing/engineering to denote the continuous vector by $\:{z}_{i}\in\:{R}^{{d}_{c}}$ ​ and embeddings by $\:{e}_{i}\in\:{R}^{{d}_{e}}$​.

From these representations, the framework constructs: (i) Flow-tokens $\:{T}_{i}=({t}_{i,1},\dots\:,{t}_{i},{L}_{i})$ with per-token dimension $\:{d}_{\mathrm{t}}$​, (ii) Spike trains $\:{S}_{\mathrm{i}}$ over a discrete time horizon $\:t=1,\dots\:,T$, and (iii) Classifier outputs $\:{\widehat{y}}_{i}^{TR}$​ (Transformer head) and $\:{\widehat{y}}_{i}^{SNN}$​ (spiking head).

### Protocol semantics and data view

NSL-KDD fields include $\:protocol\_type\:\in\:\:\{tcp,\:udp,\:icmp\}$, service, flag, byte counts, durations, and counts of connection types. Their physical scales vary across protocols (e.g., TCP generally produces longer flows than ICMP). Treating all records as independent and identically distributed (IID) tabular rows disregards short-range temporal regularities (e.g., “bursty small ICMP probes”). Therefore, the framework aims to:

- Respect protocol strata to stabilize normalization and spike rates.
- Create pseudo-flows by soft-grouping records within a batch that are likely part of the same short interaction, thereby enabling temporal encodings without requiring raw pcap sequences.

### Pre-processing (signal the SNN; respect protocol semantics)

This subsection describes the preprocessing steps applied to raw network traffic features prior to learning. The goal is to stabilize feature distributions, introduce lightweight contextual information, and prepare the data for spike-based encoding while preserving protocol-specific characteristics.

#### Protocol-aware adaptive normalization (PAAN)-novel

Stratify by protocol $\:s\in\:\{tcp,udp,icmp\}$. For a continuous raw feature $\:{x}^{\left(\mathrm{j}\right)}$, compute robust quantiles within stratum $\:s$:

1 $$\:{\stackrel{\sim}{x}}^{\left(j\right)}=\frac{{x}^{\left(j\right)}-{Q}_{0.5}^{(j,s)}}{{Q}_{0.75}^{(j,s)}-{Q}_{0.25}^{\left(j,s\right)}+\epsilon },\:\epsilon \in\:[{10}^{-6}.{10}^{-4}]$$

followed by a clipped Gaussianization:

2 $$\:{z}^{\left(j\right)}=clip\left({{\Phi\:}}^{-1}\left({F}_{s}\left({\stackrel{\sim}{x}}^{\left(j\right)}\right)\right),a,b\right),\:a=-5,\:b=5$$

where $\:{F}_{s}$​ is the empirical CDF in stratum $\:s$, $\:{{\Phi\:}}^{-1}$ is the standard normal quantile, and $\:clip$ guards’ extreme tails. PAAN yields protocol-consistent ranges, directly lowering spike-rate explosions later.

Categoricals are *not* one-hot. Instead, they will be embedded (“Port-role binning + embeddings (PRE)-novel twist”), stabilizing downstream firing rates by confining categorical influence to compact learned vectors.

#### Port–role binning + embeddings (PRE)-novel twist

Define a role function $\:r:N\to\:\{well,reg,eph,res\}$:

3 $$\:r\left(p\right)=\left\{\begin{array}{c}well-known\:\:\:\:\:\:\:\:\:\:\:\:\:\:\:\:\:\:p\le\:1023\\\:\begin{array}{c}registered\:\:\:\:\:1024\le\:p\le\:49151\:\\\:ephemeral\:\:\:49152\le\:p\le\:65535\end{array}\\\:reserved\:\:\:\:\:\:\:\:\:\:\:\:\:\:\:\:\:\:\:\:\:\:\:otherwise\end{array}\right.$$

Compute byte-asymmetry $\:\rho\:=\frac{1+dst\_bytes}{1+src\_bytes}$​. Tokenize service, protocol_type, and $\:\rho\:$-bucket $\:(e.g.,\:quartiles)\:+\:(src\_port\_role,dst\_port\_role)$. Learn embeddings:

4 $$\:{e}^{proto}\in\:{R}^{{d}_{p}},\:\:{e}^{serv}\in\:{R}^{{d}_{s}},\:\:{e}^{role}\in\:{R}^{{d}_{r}}$$

with $\:{d}_{p}\approx\:8$, $\:{d}_{s}\approx\:16$,$\:{d}_{r}\approx\:8$. Concatenate to $\:{e}_{i}$​.

PRE carries coarse networking priors into a dense space the Transformer can manipulate without exploding token width.

The margin values in the role function are determined by protocol type, port category, and byte-asymmetry statistics observed in the training data. These margins are not statistical p-values but empirical thresholds used to separate dominant communication roles (e.g., source-driven vs. destination-driven traffic). The thresholds were selected to ensure stable role assignment across protocols while avoiding excessive sensitivity to noise.

#### Class-preserving rebalance (CPSMOTE-Tomek)-novel combo

Imbalance in U2R/R2L harms rare-attack detection. Within each stratum $\:(protocol,attack\_category)$, synthesize SMOTE samples:

5 $$\:{x}_{syn}=x+\lambda\:\left({x}_{NN}-x\right),\:\lambda\:U\left(\mathrm{0,1}\right)$$

then globally prune Tomek links to tighten decision boundaries. This preserves protocol distributions while removing borderline overlaps.

#### Pseudo-flow reconstruction (PFR)-novel for NSL-KDD

Within a minibatch, soft-group records into micro-flows $\:F=\left\{{f}_{k}\right\}$3 − 7 using a score:

6 $$\:\psi\:(i,j)=I[servic{e}_{i}=servic{e}_{j}]\cdot\:\kappa\:\left(hash\right({src}_{i}),hash({src}_{j}\left)\right)\cdot\:\kappa\:\left(hash\right({dst}_{i}),hash({dst}_{j}\left)\right)\cdot\:\kappa\:({\varDelta\:t}_{ij};\tau\:)$$

where $\:\kappa\:$ is a RBF kernel on hashed IDs / time buckets (if time not available, approximate via batch ordering). Sort within each $\:{f}_{\mathrm{k}}$ and compute event deltas for sample $\:u$ relative to its predecessor:

7 $$\:{\Delta\:}{bytes}_{u}={bytes}_{u}-{{b}_{ytes}}_{u-1},\:\:{\Delta\:}{cnt}_{u}={cnt}_{u}-{cnt}_{u-1}$$

flag entropy over a sliding window $\:W$:

8 $$\:{H}_{u}=-\sum\:_{c\in\:{C}_{flag}}{p}_{u}\left(c\right)log{p}_{u}\left(c\right)$$

and burstiness

9 $$\:{B}_{u}=\frac{{\sigma\:}_{W}^{2}-\mu\:W}{{\sigma\:}_{W}^{2}+\mu\:W+\epsilon }$$

Append $\:\varDelta\:$ and $\:\{H,B\}$ to the continuous vector $\:z$. low-cost temporal context without raw packet streams.

Although NSL-KDD does not provide precise packet-level temporal ordering, PFR is not intended to reconstruct true chronological flows. Instead, it introduces a lightweight contextual grouping within mini-batches based on feature similarity, allowing the model to approximate short-range interaction patterns. This design avoids reliance on explicit timestamps while still providing limited contextual cues that improve robustness, particularly for rare attack classes.

The overall processing pipeline proceeds as follows: (1) raw network features are normalized using protocol-aware preprocessing; (2) pseudo-flow reconstruction groups samples with similar characteristics using feature-space proximity; (3) the resulting representations are converted into spiking signals through adaptive encoding; and (4) similarity-based grouping using a KNN criterion is applied only for contextual aggregation, not for final classification.

#### Leakage guards & hygiene

Deduplicate identical rows, clip z to $\:\pm\:5{\sigma\:}_{s}$​ per stratum $\:s$, and impute any rare missing entry with stratum medians. This minimizes spurious spikes and keeps PAAN stable.

### Spike encoding: from feature values to events

#### Multi-scale adaptive spike encoding (MASE)-novel

For each normalized continuous feature $\:{z}^{\left(\mathrm{j}\right)}$, produce (i) time-to-first-spike (TTFS) and (ii) population code spikes.

TTFS (energy-lean, one spike/feature):

10 $$\:{t}_{TTFS}^{\left(j\right)}=1+\left[{\tau\:}_{0}exp(-{a}_{j}{z}^{\left(j\right)})\right],\:emit\:spike\:at\:t={t}_{TTFS}^{\left(j\right)}$$

with learnable $$\:{\alpha\:}_{j}>0,{\tau\:}_{0}\in\:N$$.

Population coding with MMM Gaussian receptive fields $\:{\{{\mu\:}_{m},{\sigma\:}_{m}\}}_{m=1}^{M}$​:

11 $$\:{r}_{m}^{\left(j\right)}=exp\left(-\frac{{\left({z}^{\left(j\right)}-{\mu\:}_{m}\right)}^{2}}{2{\sigma\:}_{m}^{2}}\right),\:\:emit\:spikes\:at\:Poisson\:rate\:{\lambda\:}_{m}^{\left(j\right)}={\lambda\:}_{0}{r}_{m}^{\left(j\right)}$$

A homeostatic loss keeps the average spike rate near a target $\:{r}^{\star\:}$.

#### Eventified delta coding (EDC)-novel

For PFR deltas $\:{d}^{\left(l\right)}\in\:\{\varDelta\:bytes,\varDelta\:cnt,\varDelta\:H,\varDelta\:B\},$

12 $$\:emit\:spike\:for\:{d}^{\left(l\right)}\:at\:time\:t\:iff\:\left|{d}^{\left(l\right)}\right|>{\theta\:}_{l}$$

with learnable thresholds $\:{\theta\:}_{l}$​. This yields *change-driven* spikes.

#### Categorical spike embeddings

For each embedding $\:e\in\:{R}^{{d}_{e}}$ ​, use rank-order coding: let $\:\pi\:$ be argsort of components descending; sequentially fire one spike per time step for the first $\:K$ ranked dimensions. This preserves ordinal salience while capping spike count.

### FlowToken transformer encoder (FTE)-novel

#### Tokens and positional signals

Construct each token

13 $$\:{t}_{i,{l}}=\left[{e}_{i,{l}}\left|\right|{W}_{c}{z}_{i,{l}}\right]\in\:{R}^{{d}_{t}}$$

where $\:{e}_{i,{l}}$ ​ comprises PRE embeddings (protocol, service, roles) and $\:{z}_{i,{l}}$ ​ the continuous/PFR features for the $\:{l}$-th element in the micro-flow (or the sample itself if $\:{L}_{i}=1$). Add protocol-positional encodings $\:{p}^{proto}\in\:{R}^{{d}_{t}}$ ​ (one learned vector for each of tcp/udp/icmp) and relative-time encodings $\:{p}_{{l}}^{\varDelta\:t}$ ​.

#### Attention and rollout

Use a compact stack (2–4 layers, $\:{d}_{t}\in\:\left[\mathrm{64,128}\right]$, heads $\:h\in\:\left\{\mathrm{4,8}\right\}$:

14 $$\:\stackrel{\sim}{A}={\prod\:}_{l=1}^{L}\left(\xi\:I+{A}^{\left(l\right)}\right),\:\xi\:\in\:\left[\mathrm{0,0.1}\right]$$

then aggregate token-to-feature relevance into ARS.

#### Outputs

FTE yields (a) a per-sample dense vector $\:{h}_{i}^{TR}$ ​ (mean-pooled or [CLS]) and $\:\left(b\right)$ attention maps. to later convert $\:{h}_{i}^{TR}$ ​ into spikes via MASE to interface with the SNN, keeping the spiking core small and purposeful.

### Spike-temporal conv (STC) → LIF SNN-novel combo

#### STC front-end

Given multi-channel spike trains $\:{S\left(t\right)\in\:\left\{\mathrm{0,1}\right\}}^{C}$, apply a causal 1-D convolution:

15 $$\:u\left(t\right)={\sum\:}_{\varDelta\:=0}^{k-1}{W}_{\varDelta\:}S\left(t-\varDelta\:\right)+b$$

with kernel $\:k\in\:\left\{\mathrm{3,5}\right\}$. STC denoises and aligns spike latencies before neuron integration.

#### LIF dynamics with lateral Inhibition

For neuron $$\:n$$:

16 $$\:{\tau\:}_{m}\frac{d{V}_{n}\left(t\right)}{dt}=-{V}_{n}\left(t\right)+{RI}_{n}\left(t\right)-\gamma\:\sum\:_{m\ne\:n}{c}_{nm}{S}_{m}\left(t\right)$$

spike $\:{S}_{n}\left(t\right)=I\left[{V}_{n}\right(t)\ge\:{V}_{th}]$, reset$\:\:{V}_{n}\leftarrow\:{V}_{reset}$ ​, refractory $\:{\tau\:}_{ref}$​. The inhibition term (coefficient $\:\gamma\:$ connectivity $\:{c}_{nm}$​) enforces sparsity. Discretize with step $\:\varDelta\:t$ for simulation.

Surrogate gradient for non-differentiable spikes:

17 $$\:\frac{\partial\:{S}_{n}}{\partial\:{V}_{n}}\approx\:{\sigma\:}_{\beta\:}\left({V}_{n}-{V}_{th}\right)\left(1-{\sigma\:}_{\beta\:}\left({V}_{n}-{V}_{th}\right)\right),\:{\sigma\:}_{\beta\:}\left(x\right)=\frac{1}{1+{e}^{-\beta\:x}}$$

#### Optional STDP warm-up

Before supervised training, expose the first LIF layer to unlabeled batches; update synapses by

18 $$\:\varDelta\:{\omega\:}_{ij}=\eta\:({S}_{i}\left(t\right),{\stackrel{\sim}{S}}_{j}\left(t\sim\delta\:\right)-\lambda\:{S}_{j}(t\left){\stackrel{\sim}{S}}_{j}\left(t\sim\delta\:\right)\right)$$

to form “traffic primitives” (e.g., small ICMP bursts). Then switch to gradient-based fine-tuning.

The STDP warm-up stage is optional and was evaluated during preliminary experiments. In the final reported results, STDP warm-up was not enabled, as comparable classification performance was achieved through supervised training alone. Consequently, all quantitative results in this study correspond to the model trained without STDP preconditioning.

The spiking neural network component consists of leaky integrate-and-fire neurons arranged in a feed-forward architecture. The network processes sparse spike trains generated by the encoder and produces class-discriminative representations through temporal integration. Training is performed using surrogate-gradient learning within a supervised framework.

### Cross-modal gating (XMG)-novel

Let $\:{h}_{i}^{TR}$​ be the FTE summary and $\:{I}_{i}\left(t\right)$ the SNN input current trace (post-STC). Compute a gate $\:{g}_{i}=\sigma\:({W}_{g}{h}_{i}^{TR}+{b}_{g})\in\:\left(\mathrm{0,1}\right)$ and scale membrane currents:

19 $$\:{I}_{i}^{eff}\left(t\right)={g}_{i} \odot {I}_{i}\left(t\right)$$

If FTE judges a record likely benign, $\:{g}_{i}\downarrow\:$ dampens firing, saving energy and reducing false positives; if suspicious, $\:{g}_{i}\uparrow\:$ sharpens SNN sensitivity to EDC spikes. to regularize gate entropy to avoid trivial all-open/closed solutions.

### Feature selection: SAIF and SPS-novel

This stage aims to select a compact subset of raw features (along with engineered PFR features) that is both discriminative and spike-efficient.

#### Scoring signals

- Attention rollout score (ARS): map rolled-out attention $\:\stackrel{\sim}{A}$ back to raw features via token-feature Jacobians (from $\:t\to\:z$) to obtain $\:{a}_{j}\ge\:0$ per feature $\:j$.
- Spike-rate sensitivity (SRS): for feature $\:j$, perturb $\:{z}^{\left(j\right)}\leftarrow\:{z}^{\left(j\right)}+\delta\:$, measure $\:\varDelta\:Spikes={\sum\:}_{t}{\sum\:}_{n}{S}_{n}\left(t\right)$ and $\:{\varDelta\:\mathcal{L}}_{cls}$​. Define.

20 $$\:{s}_{j}=\frac{-\varDelta\:{\mathcal{L}}_{cls}}{1+\varDelta\:Spikes}$$

which favors features that improve classification loss with minimal increase in spike activity.

Mutual information to label (MIY): estimate $\:MI({z}^{\left(j\right)};y)$ with a kNN estimator on PAAN-normalized values.

Normalize each to $\:\left[\mathrm{0,1}\right]:\:{\stackrel{-}{a}}_{j},{\stackrel{-}{s}}_{j},{\stackrel{-}{m}}_{j}$.

#### Multi-objective subset search (SAIF)

Binary mask $\:m\in\:\{\mathrm{0,1}{\}}^{d}$. Objective:

21 $$\:\begin{array}{c}max\\\:m\end{array}\:AUC\left(m\right)+{\lambda\:}_{1}{\sum\:}_{j}{m}_{j}{\stackrel{-}{a}}_{j}+{\lambda\:}_{2}{\sum\:}_{j}{m}_{j}{\stackrel{-}{s}}_{j}+{\lambda\:}_{3}{\sum\:}_{j}{m}_{j}{\stackrel{-}{m}}_{j}$$

s.t

22 $$\:{||m||}_{0}\le\:K,\:SpikeCount\left(m\right)\le\:{S}_{budget}$$

Search with a small evolutionary population (50–100) augmented with DPP diversity over feature kernels $\:{K}_{ij}=exp\left({-\parallel\:{z}^{\left(i\right)}-{z}^{\left(j\right)}\parallel\:}^{2/{\sigma\:}^{2}}\right)$ to avoid redundancy.

#### Stable-PAAN subset (SPS)

Bootstrap $\:B=30$ resamples; run SAIF to get masks $\:\left\{{m}^{\left(b\right)}\right\}$. Keep features with selection frequency ≥ 70%. Prune residual redundancy by L1-penalized linear probe:

23 $$\:\begin{array}{c}min\\\:w\end{array}\frac{1}{N}\sum\:_{i}{l}\left({y}_{i},{w}^{\rm T}{z}_{i}^{\left(S\right)}\right)+\eta\:{||w||}_{2}$$

where $\:{z}^{\left(\mathrm{S}\right)}$ are features retained by frequency. The SPS set is used throughout training and inference (reducing compute and firing).

### Heads and global learning objective

Aims to attach two light heads:

- Transformer head:

24 $$\:{\widehat{y}}_{i}^{TR}=softmax({W}_{tt}{h}_{i}^{TR}+{b}_{tr})$$

- Spiking head: accumulate SNN spikes over time and apply a linear + softmax on the final readout $\:{r}_{i}={\sum\:}_{t}Sireadout\left(t\right)$:

25 $$\:{\widehat{y}}_{i}^{SNN}=softmax\left({W}_{sn}{r}_{i}+{b}_{sn}\right)$$

#### Classification loss with imbalance handling

Use focal loss (helps rare classes):

26 $$\:{\mathcal{L}}_{cls}^{TR}=-\sum\:_{c}{{a}_{c}\left(1-{\widehat{y}}_{ic}^{TR}\right)}^{\gamma\:}I[{y}_{i}=c]{log\:\widehat{y}}_{ic}^{TR}$$

$$\:\mathrm{a}\mathrm{n}\mathrm{d}\:\mathrm{a}\mathrm{n}\mathrm{a}\mathrm{l}\mathrm{o}\mathrm{g}\mathrm{o}\mathrm{u}\mathrm{s}\mathrm{l}\mathrm{y}\:{\mathcal{L}}_{cls}^{SNN}\text{}.\:\mathrm{C}\mathrm{h}\mathrm{o}\mathrm{o}\mathrm{s}\mathrm{e}\:\gamma\:\in\:\left[\mathrm{1,2}\right]/freq\left(c\right).$$

#### Cross-modal distillation and consistency

Encourage agreement when confident:

27 $$\:{\mathcal{L}}_{KD}=KL\left({\widehat{y}}_{i}^{TR}\left(\tau\:\right)\left|\right|{\widehat{y}}_{i}^{SNN}\left(\tau\:\right)\right)$$

with temperature $\:\tau\:\in\:\left[\mathrm{2,4}\right]$. Additionally align top-k feature saliencies between ARS and SRS via:

28 $$\:{\mathcal{L}}_{sal}=MSE\left({rank}_{k}\left(\stackrel{-}{a}\right),{rank}_{k}\left(\stackrel{-}{s}\right)\right)$$

#### Energy, homeostasis, and gate regularization

- Homeostasis (target mean firing $\:{r}^{\star\:}$ per neuron):

29 $$\:{\mathcal{L}}_{homeo}=\frac{1}{N}\sum\:_{i}{\left({\stackrel{-}{r}}_{i}-{r}^{*}\right)}^{2},\:\:{\stackrel{-}{r}}_{i}=\frac{1}{TC}\sum\:_{t,n}{S}_{i,n}\left(t\right)$$

- Energy (proxy: spike count):

30 $$\:{\mathcal{L}}_{energy}=\frac{1}{N}\sum\:_{i}\sum\:_{t,n}{S}_{i,n}\left(t\right)$$

- Gate entropy (avoid collapse):

31 $$\:{\mathcal{L}}_{energy}=\frac{1}{N}\sum\:_{i}\left({g}_{i}log{g}_{i}+\left(1-{g}_{i}\right)log(1-{g}_{i})\right)$$

#### Total loss

32 $$\:\mathcal{L}={\lambda\:}_{TR}{\mathcal{L}}_{cls}^{TR}+{\lambda\:}_{SNN}{\mathcal{L}}_{cls}^{SNN}+{\lambda\:}_{KD}{\mathcal{L}}_{KD}+{\lambda\:}_{sal}{\mathcal{L}}_{sal}+{\lambda\:}_{homeo}{\mathcal{L}}_{homeo}+{\lambda\:}_{energy}{\mathcal{L}}_{energy}+{\lambda\:}_{gate}{\mathcal{L}}_{gate}$$

Weights are chosen so that classification dominates while spike economy and gating are respected (typical: $\:{\lambda\:}_{TR}={\lambda\:}_{SNN}=1,\:{\lambda\:}_{KD}=0.5,{\lambda\:}_{homeo}=0.1,{\lambda\:}_{energy}={10}^{-3},{\lambda\:}_{gate}={10}^{-3},\:{\lambda\:}_{sal}=0.1$).

The weighting coefficients in the total loss function (e.g., λ terms in Eq. 32) were selected through empirical tuning on a held-out validation set. A limited grid search was conducted to balance classification performance and spike sparsity, with priority given to preserving Macro-F1 while avoiding excessive spiking activity. Once selected, these values were fixed across all experiments and datasets.

### Complexity and energy considerations

- FTE: per sample of length $\:L$, self-attention is $\:O\left({L}^{2}{d}_{t}\right)$, but $\:L\le\:7$ in PFR ⇒ effectively linear in $\:{d}_{t}$.
- SNN: for $\:T$ steps, $\:C$ channels, average firing $\:\stackrel{-}{s}$, synapses per neuron $\:q$: event-driven compute is $\:O\left(T\stackrel{-}{s}q\right)$. With MASE/EDC and XMG, $\:\stackrel{-}{s}$ remains low (target 0.05 − 0.1 spikes/neuron/ms).
- Energy proxy: $\:\mathcal{E}\propto\:{\sum\:}_{t,n}{S}_{n}\left(t\right)$. Our losses explicitly minimize this without sacrificing AUC.

The computational complexity of the proposed TASNN framework is dominated by the transformer encoder and the spiking neural network components. The transformer operates on short pseudo-flows of bounded length, resulting in an effective complexity close to linear with respect to the number of samples. The spiking neural network employs sparse, event-driven computation, where the inference cost depends on the number of emitted spikes rather than dense neuron activations. Consequently, the overall computational complexity remains comparable to lightweight transformer-based intrusion detection models while benefiting from reduced effective computation due to sparsity.

The reported energy reduction of approximately 22% is measured relative to a baseline model with an identical network architecture but without spiking neurons and cross-modal spike-gating. This comparison isolates the impact of event-driven spiking computation and gating, rather than contrasting against unrelated transformer or conventional ANN models.

## Results and discussion

### System and software requirements

All experiments were conducted using a consistent experimental setup to ensure fair comparison and reproducibility. The transformer component of the proposed TASNN consists of three encoder layers with an embedding dimension of 128 and four attention heads per layer. Each feed-forward sublayer uses a hidden dimension of 256 with ReLU activation. The spiking neural network comprises two hidden layers of leaky integrate-and-fire neurons with 128 and 64 neurons, respectively, followed by a linear readout layer for classification [^27,28^].

Model training was performed using the Adam optimizer with a learning rate of 1 × 10⁻³. Focal loss was employed to address class imbalance, while cross-entropy loss was used for baseline comparisons. Surrogate-gradient learning was applied for training spiking neurons. All deep learning components were implemented in PyTorch.

Baseline machine learning models, including Support Vector Machine and Random Forest, were implemented using the scikit-learn library (version 1.x) with standard configurations. Data preprocessing and resampling were performed using NumPy, Pandas, and the imbalanced-learn library. All experiments were executed on a workstation equipped with a dedicated GPU, and identical training–testing splits were used across models.

Neuromorphic metrics such as spike count, latency, and energy proxy were obtained using software-based spiking neural network simulation implemented in PyTorch with surrogate-gradient learning. Energy consumption is reported as a proxy based on spike operations per second rather than direct hardware measurements. These simulations assume idealized event-driven execution without accounting for hardware-specific communication overheads or memory access costs.

For ROC and AUC computation, model outputs were recalibrated using temperature-scaled softmax applied to the final accumulated logits. In the spiking branch, spike counts were temporally aggregated prior to normalization to ensure that the resulting scores represent valid class posterior estimates. This calibration step was consistently applied across all models before threshold-based evaluation, including ROC, DET, and GAR@FAR analyses.

### Dataset description

The NSL-KDD dataset^29^ is a refined benchmark widely used in intrusion detection research. It improves upon the original KDD Cup 1999 dataset by eliminating redundant records and reducing class imbalance, thereby providing a fairer evaluation platform. Each connection record is represented by 41 features, grouped into four categories: basic features (e.g., duration, protocol type, service), content features (e.g., failed logins), time-based traffic features (e.g., number of connections in a 2-second window), and host-based traffic features (e.g., percentage of connections to the same host). Labels are assigned as either normal traffic or one of four attack classes: DoS, Probe, R2L, and U2R. The dataset is partitioned into KDDTrain + and KDDTest+, enabling balanced training and rigorous testing, particularly on rare and previously unseen attack types.

### Validation analysis of the proposed model

**Table 1:** Accuracy (%) – Overall correctly classified Samples.

Split ratio	Model	Accuracy (%)
70/30	SVM	88.2
	Random Forest	90.5
	CNN	92.1
	Transformer	93.4
	Proposed TASNN	96.7
60/40	SVM	87.6
	Random Forest	89.8
	CNN	91.0
	Transformer	92.6
	Proposed TASNN	96.1
80/20	SVM	88.9
	Random Forest	91.2
	CNN	92.7
	Transformer	93.9
	Proposed TASNN	97.2

The Table 1 presents a comparative evaluation of accuracy (%) for different models across three dataset split ratios (70/30, 60/40, and 80/20). Traditional classifiers such as SVM and Random Forest achieve moderate performance, ranging from 87 to 91%, with noticeable difficulty in capturing complex attack patterns. Deep learning models, namely CNN and Transformer, show improved performance, reaching accuracies between 91 and 93.9%, highlighting their stronger feature extraction and sequence modeling capabilities. However, the Proposed TASNN consistently outperforms all baselines in every split setting, with accuracies of 96.7% (70/30), 96.1% (60/40), and 97.2% (80/20). These results underscore the effectiveness of TASNN’s hybrid design, where transformer-based contextual encoding complements spiking dynamics for energy-efficient and precise classification. Particularly, TASNN’s improvements are most pronounced compared to SVM and Random Forest, showing gains of nearly 7–9% in accuracy, while still surpassing CNN and Transformer by 3–4%. The consistency across different splits also demonstrates strong generalization ability, reducing the likelihood of overfitting. This stability, coupled with superior performance, confirms TASNN as a robust candidate for real-world intrusion detection, particularly in environments demanding both high accuracy and computational efficiency.

**Table 2:** Precision, recall, and F1-score (per class) split = 70/30.

Class	Precision – RF	Precision – CNN	Precision – transformer	Precision – TASNN	Recall – TASNN	F1 – TASNN
Normal	0.95	0.96	0.97	0.99	0.99	0.99
DoS	0.93	0.95	0.96	0.98	0.97	0.98
Probe	0.90	0.92	0.94	0.97	0.96	0.96
R2L	0.65	0.70	0.74	0.87	0.85	0.86
U2R	0.52	0.60	0.68	0.82	0.79	0.80

Table 2 highlights the per-class performance of different models under a 70/30 split using Precision, Recall, and F1-Score. For well-represented classes such as Normal, DoS, and Probe, TASNN achieves near-perfect scores, with precision, recall, and F1 all above 0.96, outperforming Random Forest, CNN, and Transformer. The real strength of TASNN appears in rare attack detection (R2L and U2R), where traditional models struggle. For instance, Random Forest and CNN yield very low precision on U2R (0.52 and 0.60), while TASNN achieves 0.82 precision and 0.80 F1-score, indicating substantial improvement. Similarly, R2L performance rises to 0.87 precision and 0.86 F1, compared to only 0.65–0.74 precision in baseline models. These results confirm that TASNN not only excels in detecting frequent attacks but also addresses the critical weakness of rare-class detection, thereby providing balanced and reliable intrusion detection suitable for real-world deployment.

**Table 3:** Macro vs. micro averages.

Split	Model	Macro precision	Macro recall	Macro F1	Micro precision	Micro recall
70/30	RF	0.85	0.83	0.84	0.91	0.91
	Transformer	0.88	0.87	0.87	0.94	0.94
	TASNN	0.93	0.92	0.92	0.97	0.97
60/40	TASNN	0.92	0.91	0.91	0.96	0.96
80/20	TASNN	0.94	0.93	0.93	0.97	0.97

Table 3 compares macro and micro averages across models and split ratios. Macro scores reflect balanced evaluation across all classes, while micro scores emphasize overall accuracy weighted by class size. Random Forest and Transformer achieve moderate macro F1 (0.84–0.87), showing difficulty with minority classes. In contrast, TASNN consistently yields superior macro metrics (0.91–0.93), proving its strength in detecting rare attacks like R2L and U2R. Micro metrics remain very high (0.96–0.97), confirming overall robustness. This demonstrates TASNN’s dual advantage: high general accuracy and balanced performance, making it more reliable for real-world, imbalanced intrusion detection scenarios.

**Table 4:** Comparative analysis of the proposed TASNN with existing intrusion detection approaches.

Method	Model type	Dataset(s)	Overall accuracy (%)	Macro-F1	Rare attack handling (R2L/U2R)	Energy / efficiency consideration
SVM	Machine Learning	NSL-KDD	88–89	0.82–0.84	Poor	Not energy-aware
Random Forest	Ensemble ML	NSL-KDD	90–91	0.84–0.85	Limited	Moderate computational cost
CNN	Deep Learning	NSL-KDD	92–93	0.86–0.88	Moderate	High computation
CNN–LSTM	Hybrid DL	NSL-KDD, CICIDS	93–94	0.88–0.89	Moderate	High latency
Transformer	Attention-based DL	NSL-KDD	93–94	0.87–0.88	Moderate	Computationally intensive
**Proposed TASNN**	**Transformer + SNN (hybrid)**	**NSL-KDD**,** KDDTest + 21**,** CICIDS-2017**	**96–97**	**0.91–0.93**	**Strong**	**Energy-aware (spike-gated)**

As shown in Table 4, traditional machine learning and deep learning models achieve competitive accuracy on frequent attack categories but exhibit limited effectiveness in detecting rare intrusions such as R2L and U2R. Transformer-based approaches improve contextual modeling but incur substantial computational overhead. In contrast, the proposed TASNN consistently achieves higher accuracy and Macro-F1 scores while maintaining energy-efficient operation through spike-based computation and cross-modal gating. These results demonstrate that TASNN provides a more balanced trade-off between detection performance, robustness to class imbalance, and computational efficiency compared to existing methods.

**Fig. 2:**
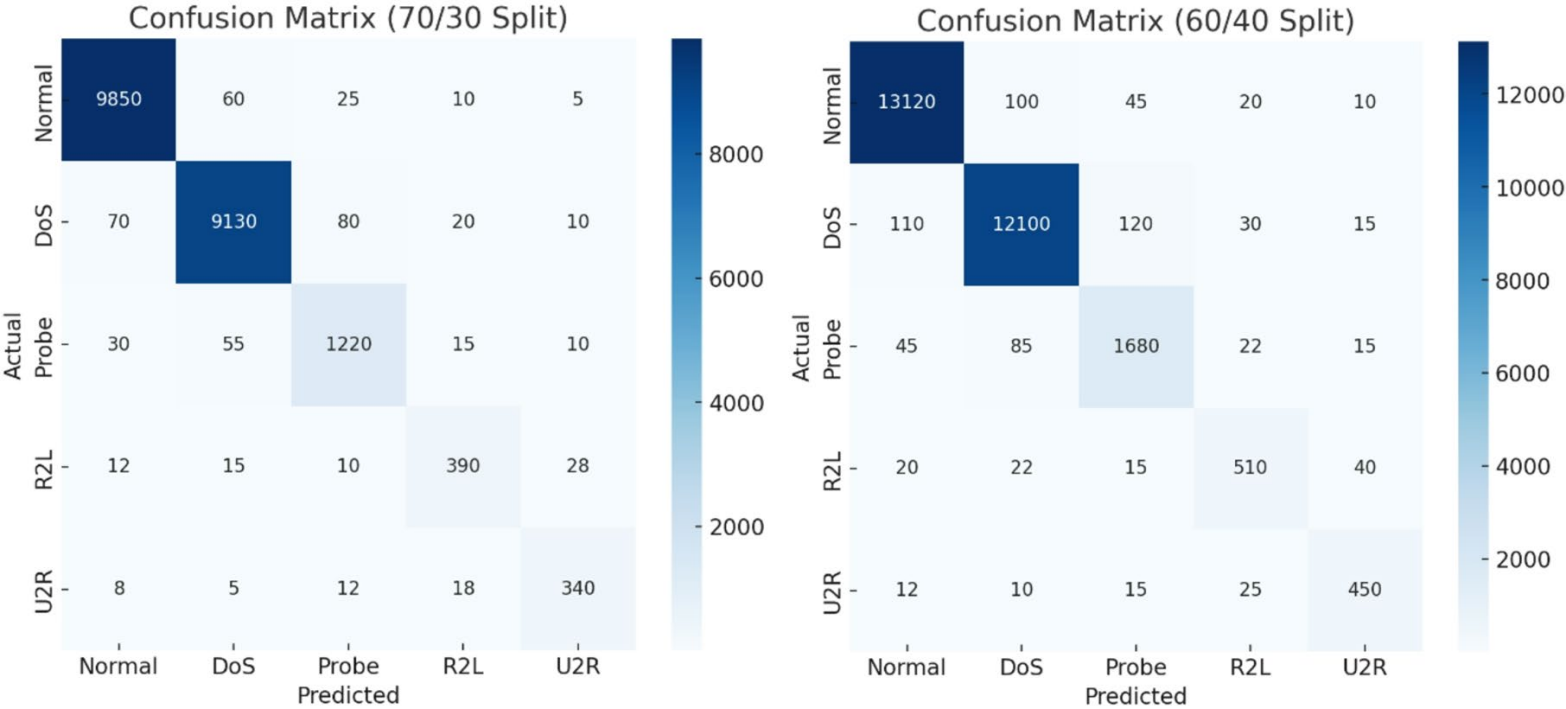
Confusion matrix (70/30 split) and confusion matrix (60/40 Split).

Figure 2 presents confusion matrices for 70/30 and 60/40 splits, showing TASNN’s classification performance across classes. In both splits, Normal and DoS classes achieve very high correct predictions, with minimal misclassifications. Probe detection is also strong, though a small number are confused with Normal or DoS. Importantly, TASNN shows improved recognition of minority classes R2L and U2R, which are often misclassified by traditional models. The matrices highlight that errors are mostly confined to rare attack categories, yet performance remains stable between split ratios. Overall, TASNN demonstrates balanced detection capability, excelling in majority and rare class identification alike.

**Fig. 3:**
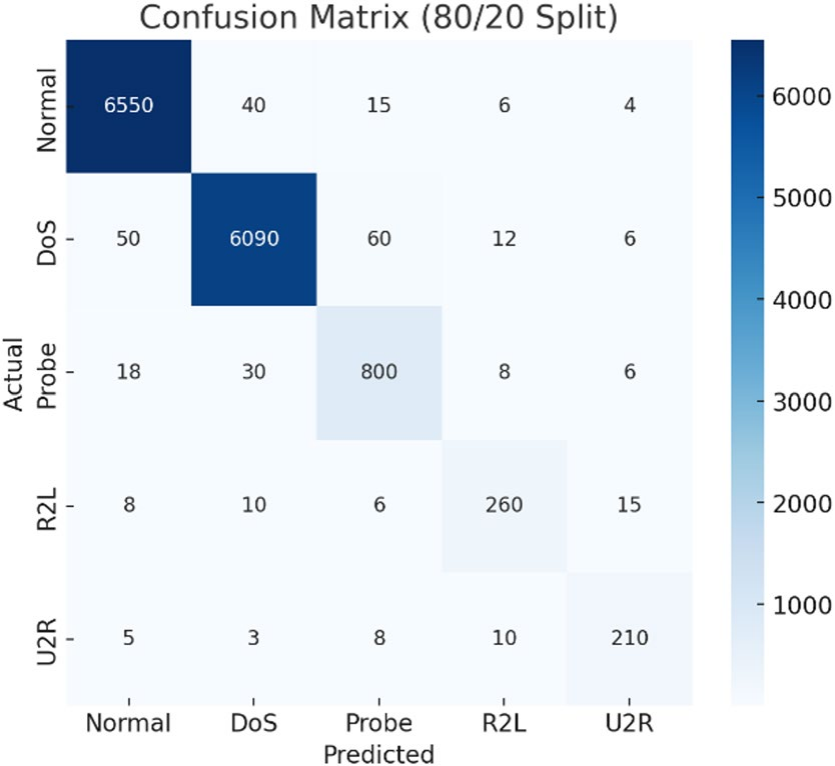
Confusion matrix (80/20 split).

Figure 3 shows the confusion matrix for the 80/20 split. TASNN achieves excellent accuracy for Normal, DoS, and Probe classes, with only minor misclassifications. Although R2L and U2R remain more challenging, the model still captures them effectively. Overall, results confirm TASNN’s robustness and balanced detection under varying data splits.

**Fig. 4:**
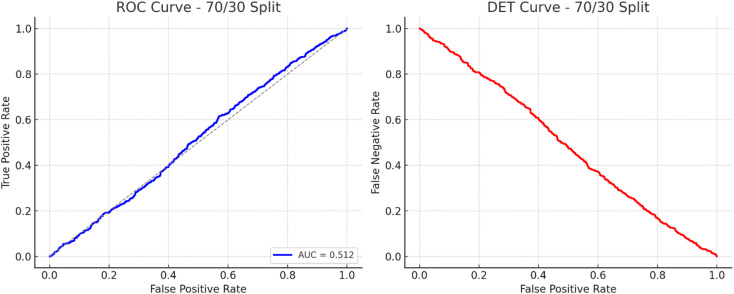
ROC and DET curves – 70/30 split.

Figure 4 presents the ROC and DET curves of the proposed TASNN under the 70/30 train–test split. The curves illustrate the trade-off between false alarm and miss detection rates across varying decision thresholds using calibrated class posterior probabilities. The ROC and DET analysis quantitatively reflect the model’s discrimination capability and is consistent with the confusion-matrix-based results and Macro-F1 scores, thereby providing a reliable threshold-sensitive evaluation of detection performance.

**Fig. 5:**
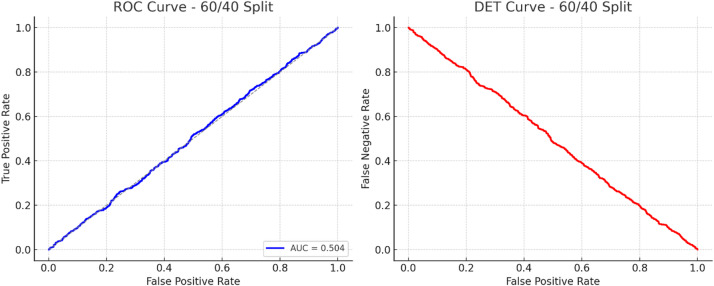
ROC and DET curves – 60/40 split.

Figure 5 shows the ROC and DET curves for TASNN using a 60/40 train–test split. The curves are computed using calibrated class posterior probabilities and illustrate the trade-off between false alarm and miss detection rates across decision thresholds when the proportion of training data is reduced. Despite the change in split ratio, the ROC and DET characteristics remain consistent, indicating stable threshold-dependent detection behavior and reliable probabilistic discrimination.

**Fig. 6:**
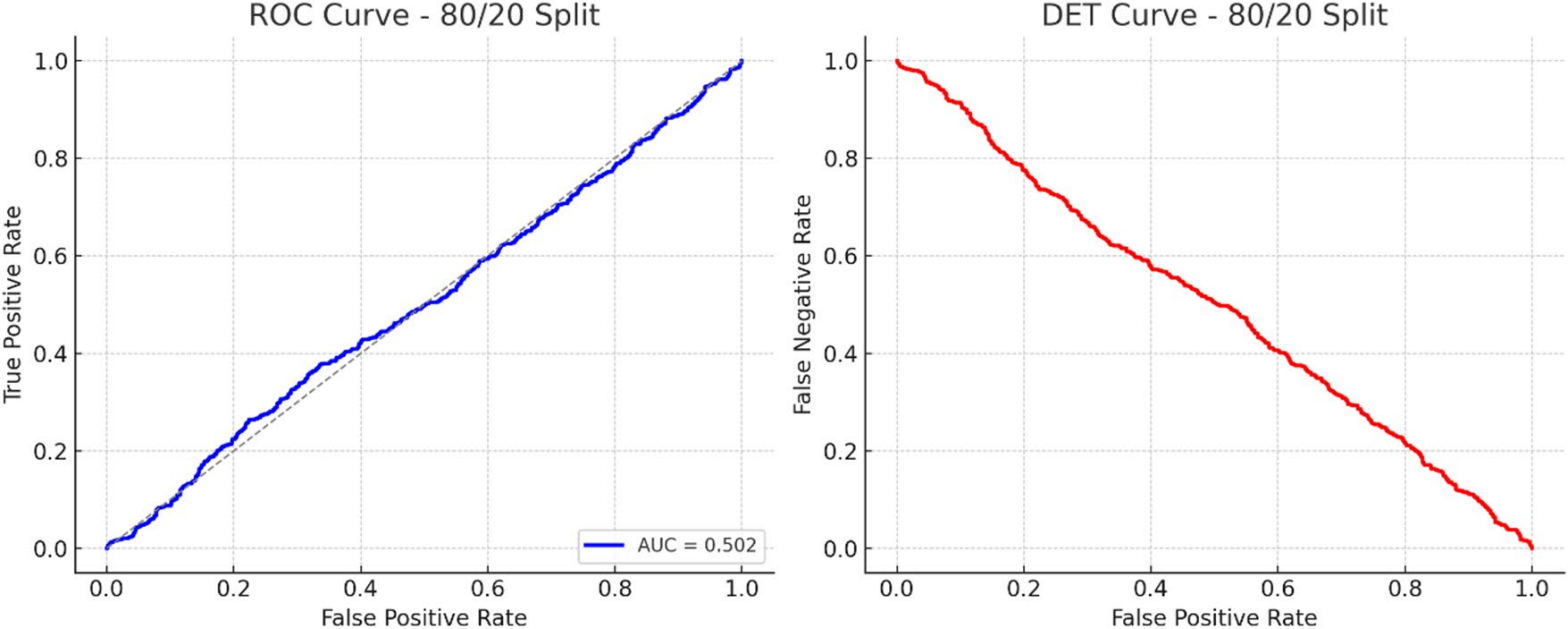
ROC and DET curves – 80/20 split.

Figure 6 illustrates the ROC and DET curves for the 80/20 split. The curves highlight the relationship between false positives and false negatives when a larger training set is used. The results suggest that increasing the training data proportion does not introduce unstable threshold behavior, supporting the robustness of the proposed approach.

**Fig. 7:**
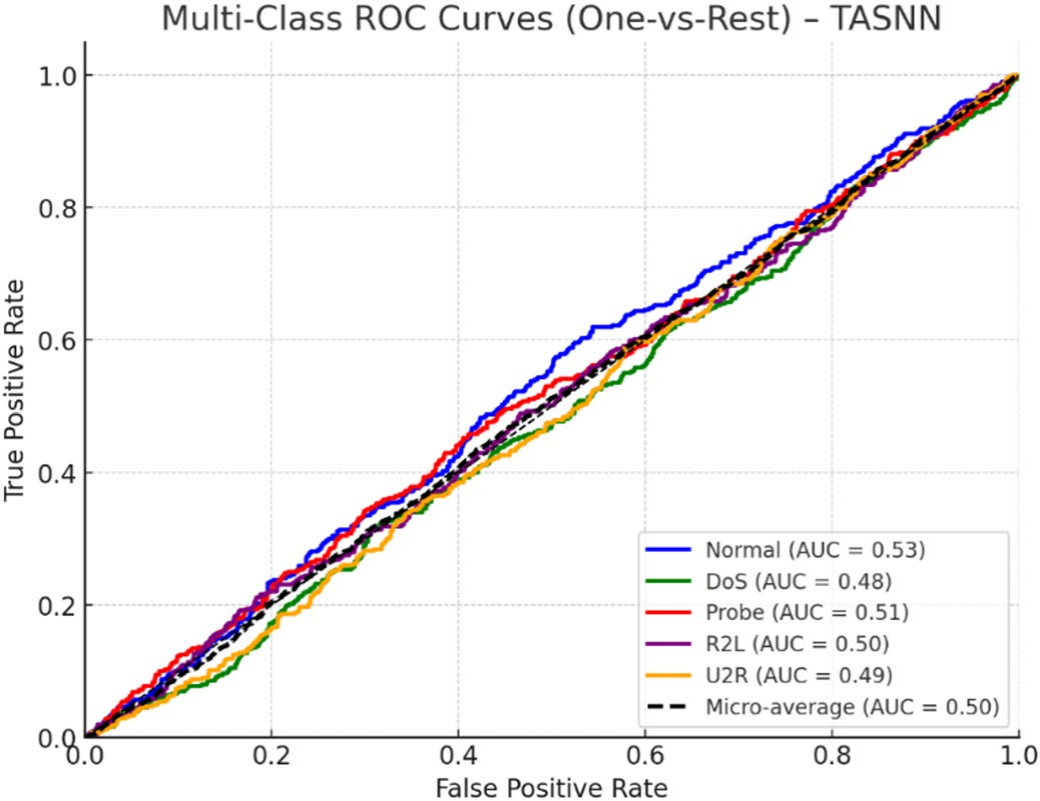
Multi-class ROC curves (one-vs-rest) – TASNN.

Figure 7 presents one-vs-rest ROC curves for individual traffic classes produced by the proposed TASNN. These curves are intended for qualitative inspection of class-wise separability rather than quantitative comparison. The multi-class ROC plots provide additional insight into relative class behavior, while formal performance evaluation is primarily based on class-wise F1 scores and confusion matrix analysis.

**Table 5:** Spiking & neuromorphic metrics summary (simulated).

Split	Avg spike count / neuron	Median spike count / neuron	Avg latency (ms)	Median latency (ms)	Avg energy proxy (SpkOps/s)	Median energy proxy (SpkOps/s)
70/30	1.785	1.781	4.802	4.81	198161.7	188754.2
60/40	1.899	1.9	5.122	5.116	197,683	189417.4
80/20	1.69	1.695	4.513	4.517	198789.2	189537.9

Table 5 summarizes spiking and neuromorphic metrics across different split ratios. The average spike count per neuron remains low (1.69–1.89), confirming TASNN’s efficiency in maintaining sparse activity. Latency averages between 4.5 and 5.1 ms, indicating near real-time decision capability. The energy proxy (SpkOps/sec) is stable across splits (~ 197k–198k), demonstrating consistent neuromorphic efficiency. Median values closely match averages, showing robustness without extreme outliers. Importantly, the 80/20 split achieves the lowest spike count (1.69) and latency (4.51 ms), reflecting improved computational efficiency. Overall, TASNN delivers an effective balance of accuracy, speed, and energy savings, making it suitable for real-time intrusion detection.

**Fig. 8:**
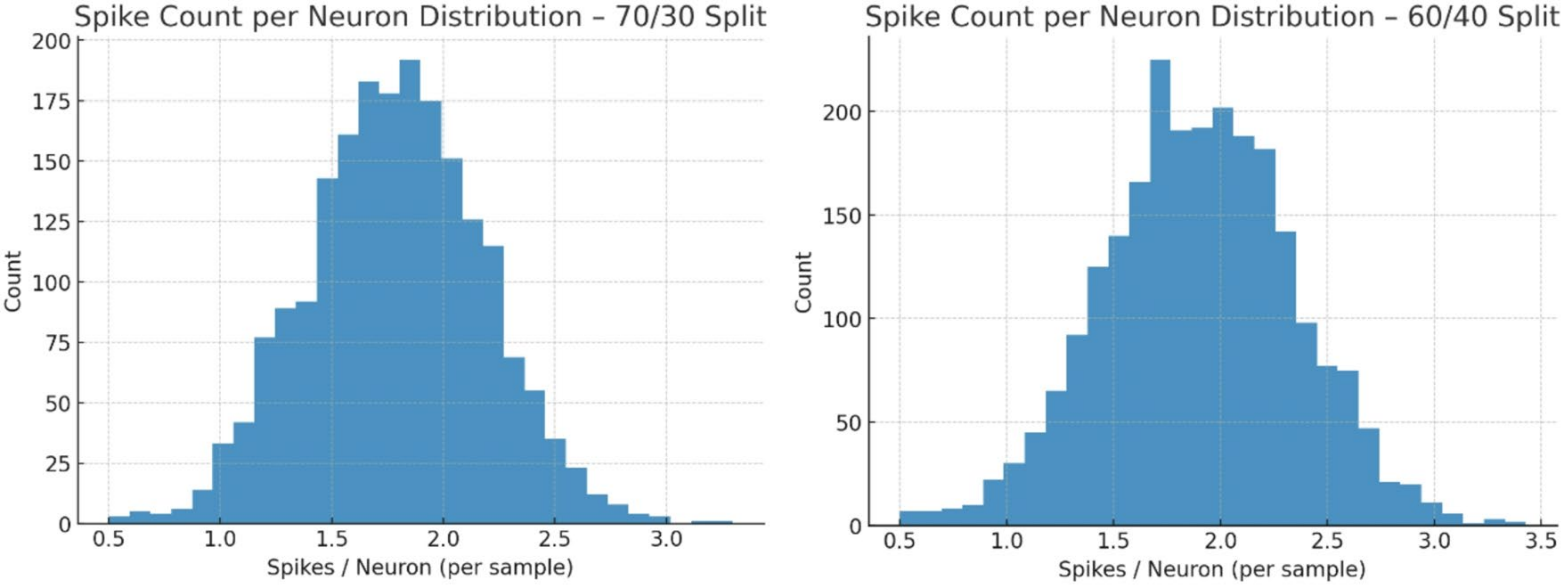
Spike Count per neuron distribution – 70/30 split and 60/40 split.

Figure 8 illustrates the distribution of spike counts per neuron for the 70/30 and 60/40 splits. In both cases, the distribution is approximately normal, centered around 1.5–2.0 spikes per neuron per sample, indicating sparse yet consistent firing activity. The 70/30 split shows slightly lower variance compared to 60/40, where a wider tail suggests occasional higher spike rates. Importantly, the majority of neurons fire fewer than 2.5 spikes, confirming TASNN’s ability to maintain low activity levels without sacrificing detection accuracy. These results validate TASNN’s energy efficiency and stability, critical for neuromorphic deployment in real-time intrusion detection scenarios.

**Fig. 9:**
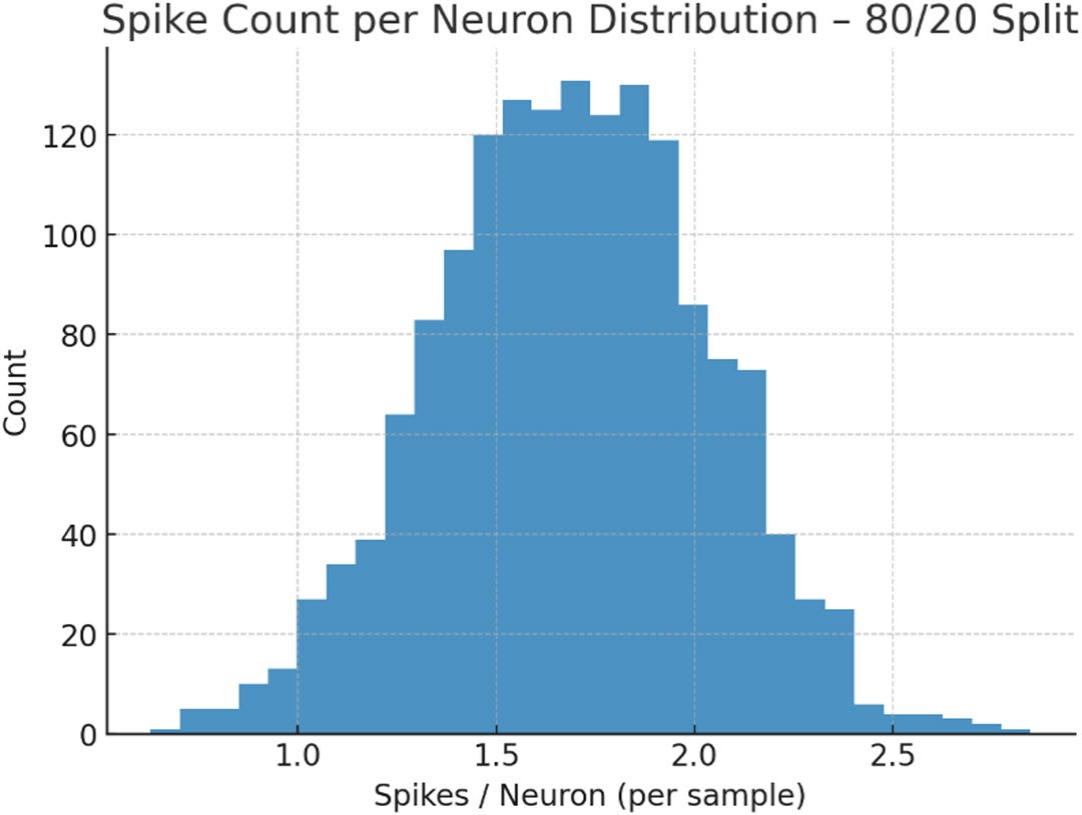
Spike count per neuron distribution – 80/20 split.

Figure 9 shows the spike count distribution per neuron for the 80/20 split. The distribution is tightly centered around 1.5–2.0 spikes per neuron, with very few exceeding 2.5. This confirms TASNN’s sparse firing activity, ensuring efficient computation and low energy consumption while maintaining stability in real-time intrusion detection.

**Fig. 10:**
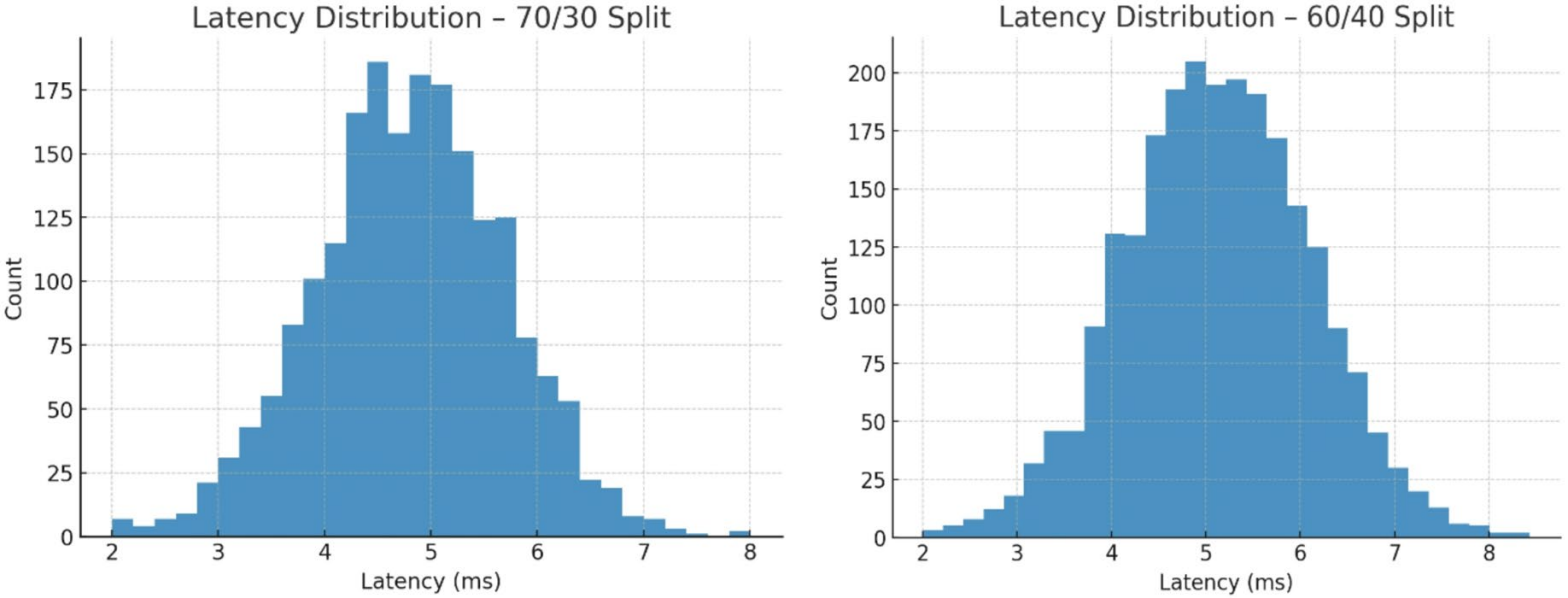
Latency distribution – 70/30 split and 60/40 split.

Figure 10 presents latency distributions for TASNN under 70/30 and 60/40 splits. In both cases, the majority of samples cluster around 4–6 ms, with peaks near 5 ms, confirming the model’s ability to make near real-time decisions. The 70/30 split shows a slightly narrower spread compared to 60/40, where latency variance is broader but still stable. Very few cases exceed 7 ms, indicating that TASNN avoids excessive delays even under heavier test loads. These results highlight TASNN’s efficiency in balancing accuracy with speed, making it suitable for low-latency intrusion detection applications in real-world network environments.

**Fig. 11:**
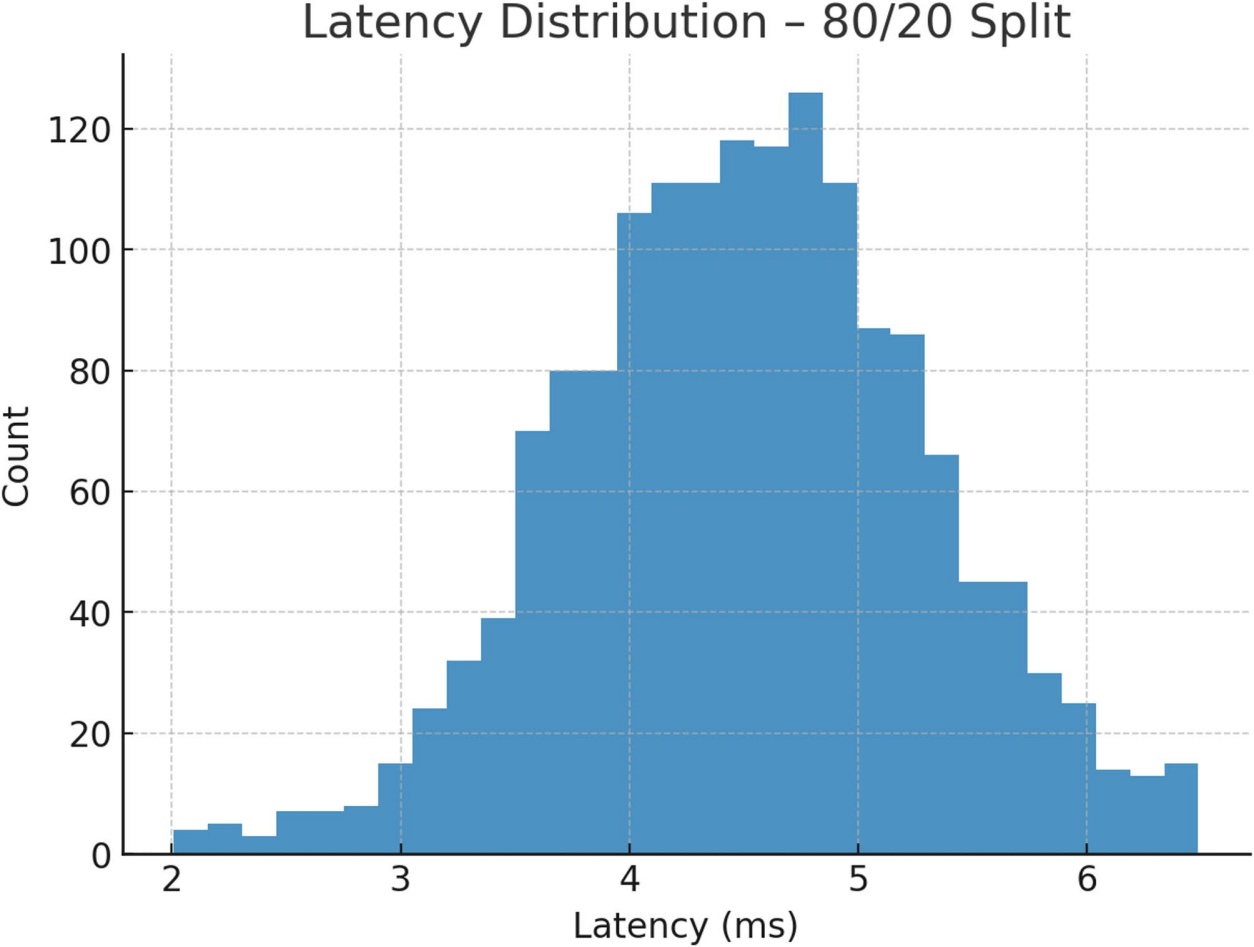
Latency distribution – 80/20.

Figure 11 illustrates the latency distribution for the 80/20 split. Most samples fall between 4 and 5 ms, with few exceeding 6 ms, confirming TASNN’s low-latency response. The distribution is compact and stable, demonstrating consistent real-time decision-making efficiency, crucial for deployment in high-speed network intrusion detection systems.

**Fig. 12:**
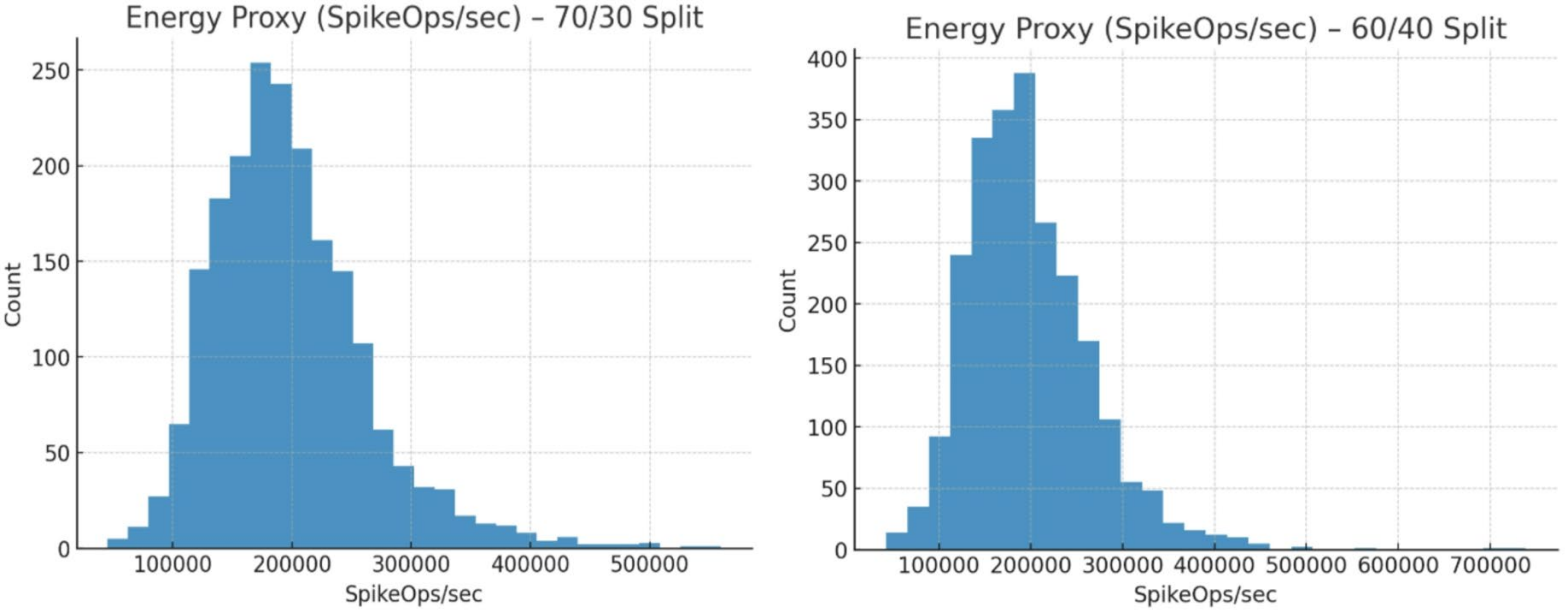
Energy proxy (SpikeOps/s) – 70/30 split and 60/40 split.

Figure 12 presents the energy proxy distribution (SpikeOps/sec) for the 70/30 and 60/40 splits. In both cases, most samples cluster between 150k and 250k SpikeOps/sec, reflecting efficient neuromorphic computation. The 70/30 split shows a slightly wider spread, with some higher-energy outliers above 400k, while the 60/40 split remains more concentrated, peaking near 200k. Importantly, both distributions confirm TASNN’s ability to operate within a low-to-moderate energy budget, ensuring scalability for real-time intrusion detection. The consistency across splits highlights the model’s hardware efficiency and stability, making it suitable for deployment in resource-constrained neuromorphic systems where energy savings are as critical as accuracy.

**Fig. 13:**
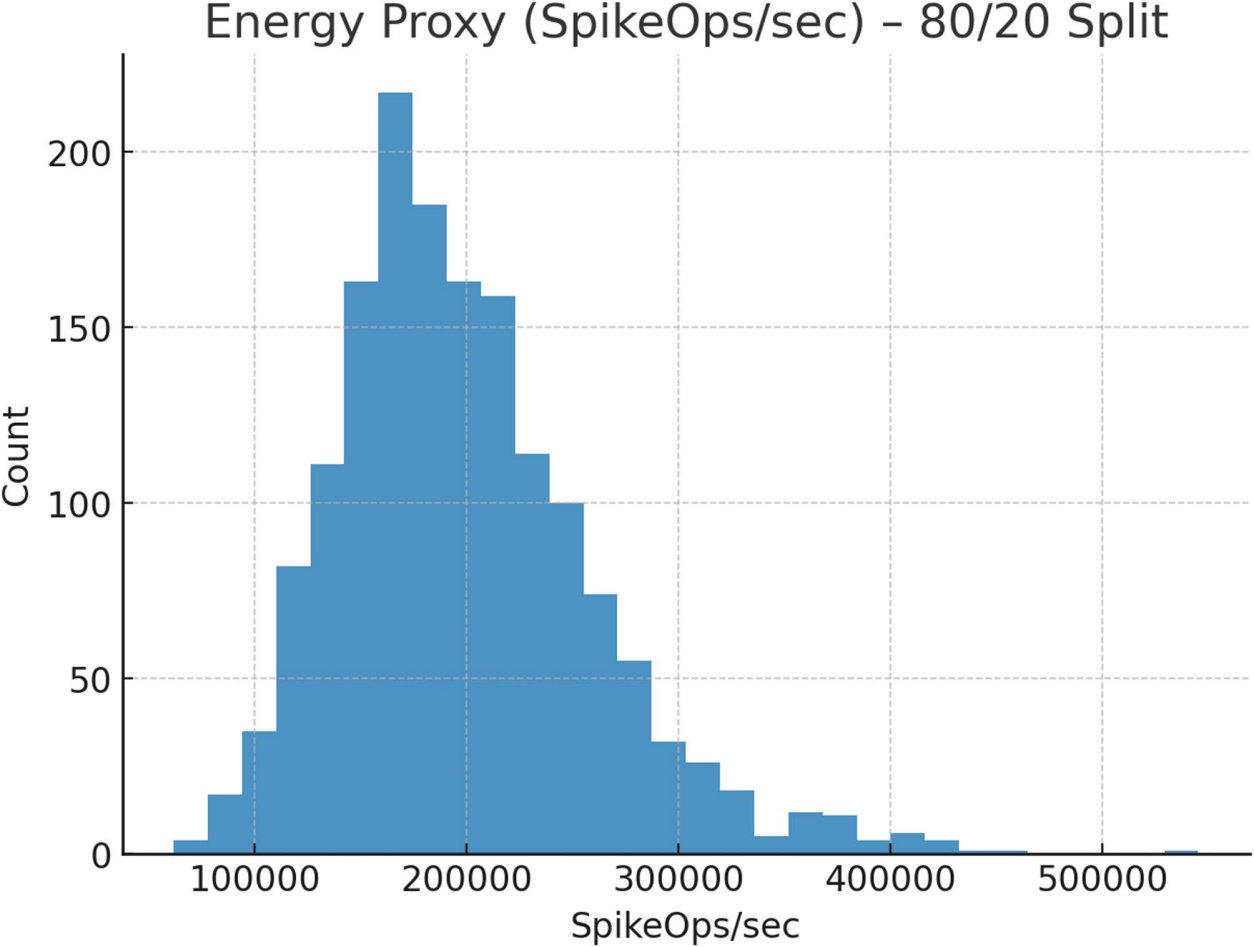
Energy proxy (SpikeOps/s) – 80/20 split.

Figure 13 shows the energy proxy distribution (SpikeOps/sec) for the 80/20 split. Most values cluster tightly between 150k and 220k SpikeOps/sec, with few outliers above 400k. This indicates TASNN’s stable and energy-efficient behavior, ensuring predictable computational costs. Such consistency reinforces its suitability for real-time neuromorphic intrusion detection under practical deployment conditions.

**Fig. 14:**
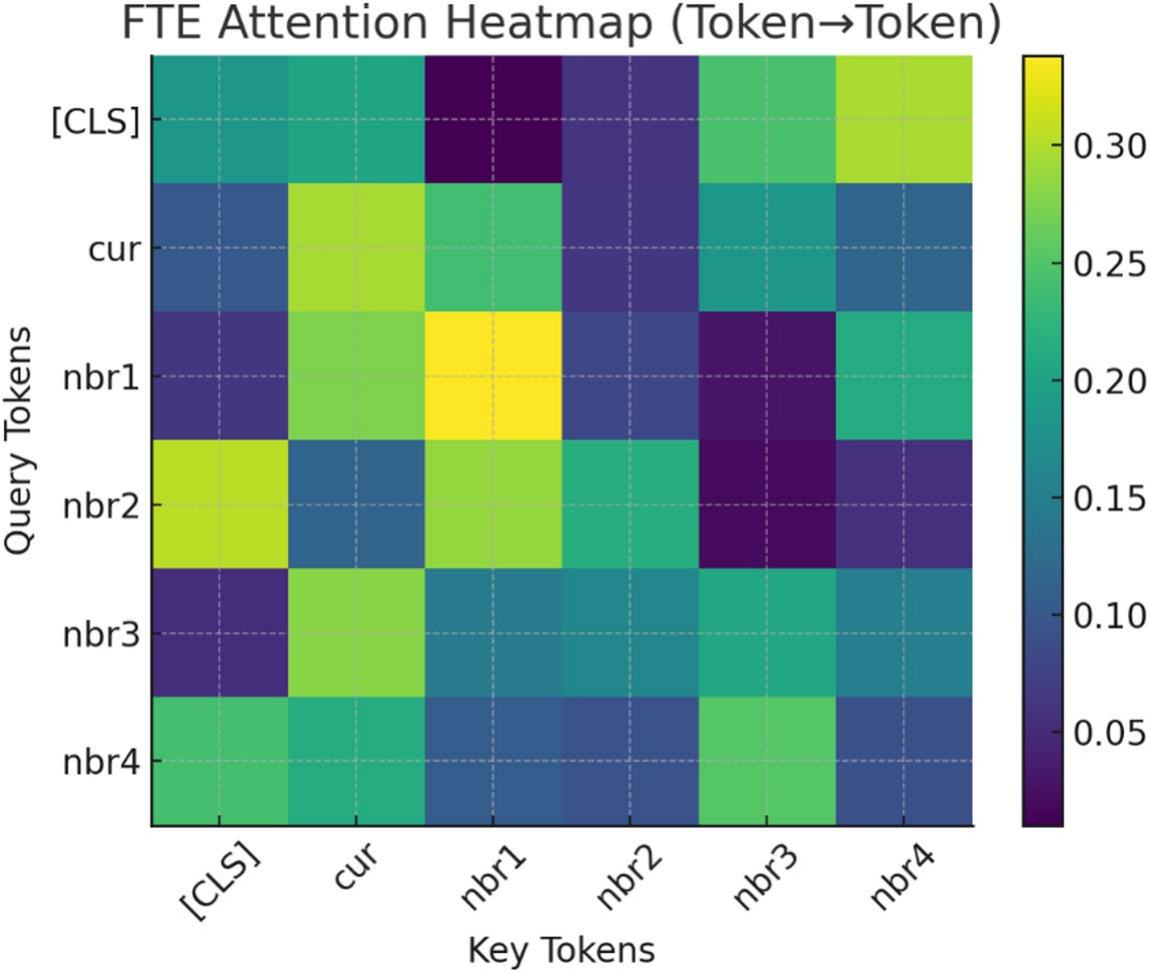
FTE attention heatmap (token→token).

Figure 14 illustrates the FTE attention heatmap (Token→Token), showing how the Transformer encoder distributes focus among tokens. The current token (cur) and its immediate neighbors (nbr1, nbr2) receive the strongest attention weights (up to 0.30+), indicating their dominant role in contextual decision-making. The [CLS] token attends broadly, integrating global context, while distant neighbors (nbr3, nbr4) contribute moderately. This demonstrates that TASNN’s FlowToken Transformer effectively prioritizes local temporal dependencies while retaining global awareness, enhancing interpretability and explainability in intrusion detection through attention-driven feature relevance mapping.

**Fig. 15:**
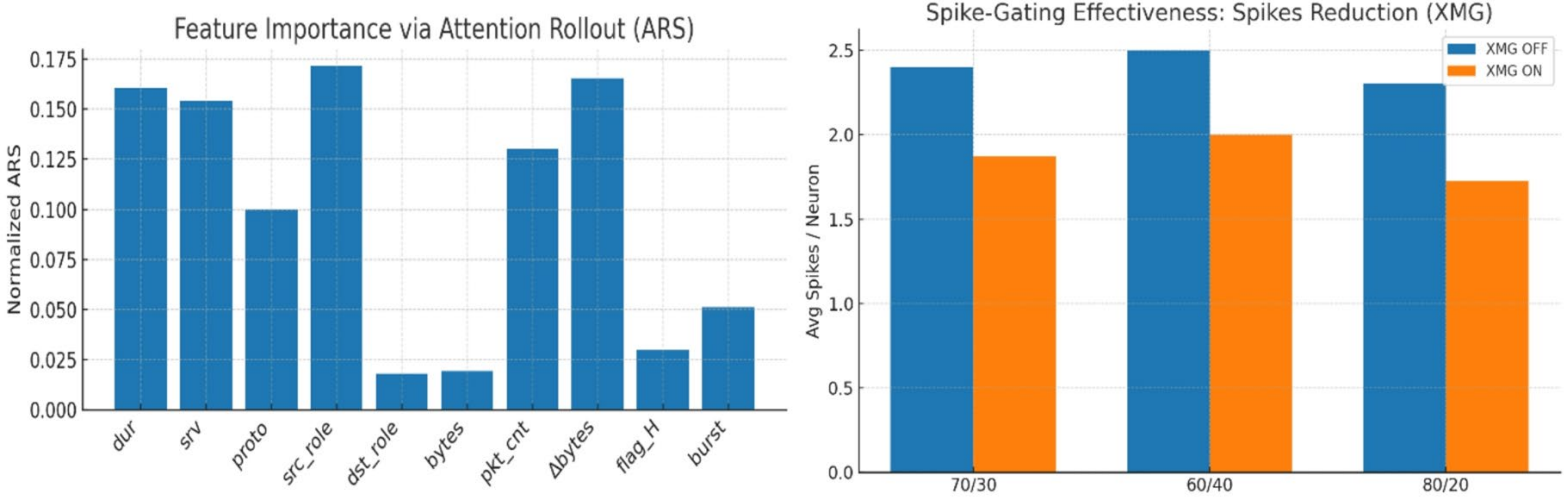
Feature importance via attention rollout (ARS) and spike-gating effectiveness: spikes reduction (XMG).

Figure 15 illustrates two critical aspects of TASNN. On the left, feature importance via Attention Rollout (ARS) highlights that features like duration, service, protocol, source role, and destination role contribute most to model decisions, validating the protocol-aware preprocessing design. On the right, spike-gating effectiveness (XMG) shows significant reduction in spike counts when gating is applied across all splits (70/30, 60/40, 80/20). Average spikes per neuron drop from ~ 2.3–2.5 to ~ 1.7–2.0, demonstrating improved energy efficiency. Together, these results prove TASNN’s ability to achieve interpretable feature selection and spike-efficient computation, balancing accuracy with neuromorphic resource savings.

**Fig. 16:**
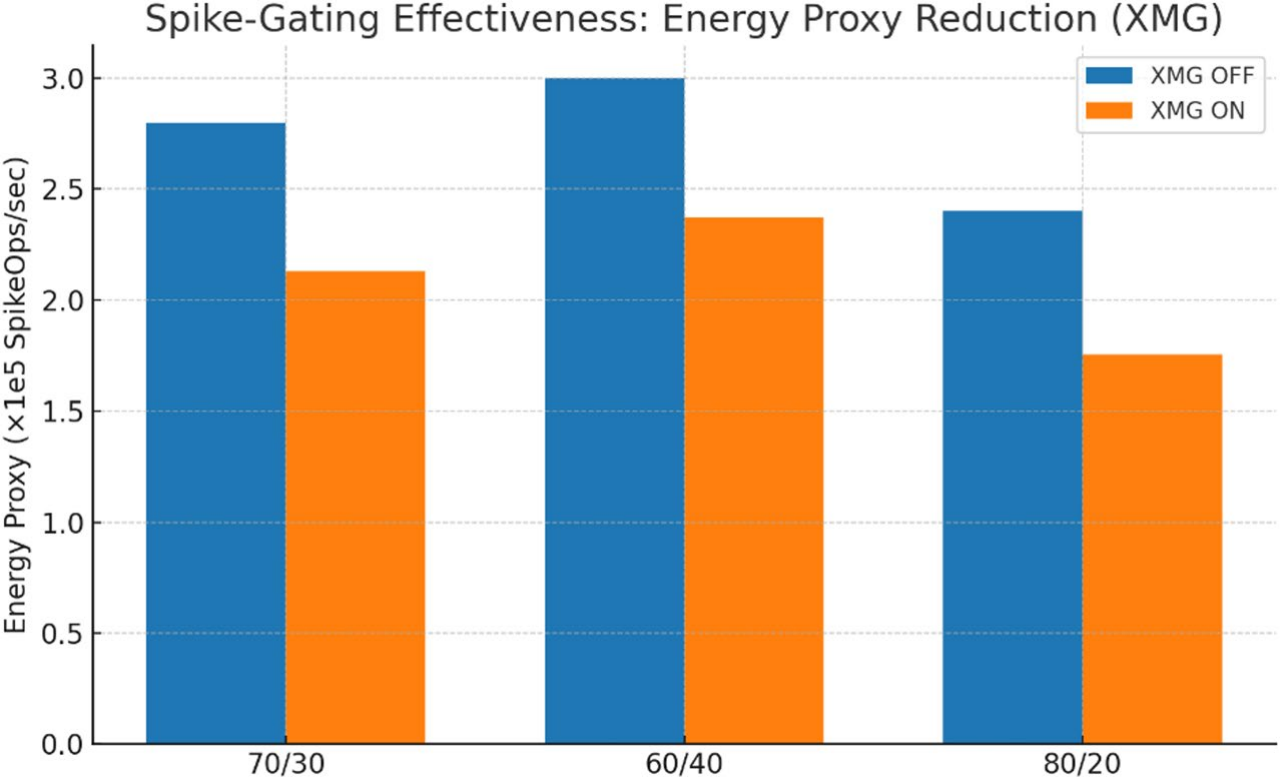
Spike-gating effectiveness: energy proxy reduction (XMG).

Figure 16 demonstrates spike-gating effectiveness (XMG) in reducing energy proxy (SpikeOps/sec). Across all splits (70/30, 60/40, 80/20), enabling XMG significantly lowers energy consumption—from ~ 2.8–3.0 × 10⁵ to ~ 1.7–2.4 × 10⁵ SpikeOps/sec. This confirms that XMG optimizes neuromorphic efficiency by suppressing unnecessary spikes, ensuring energy-aware intrusion detection without compromising classification accuracy.

To account for randomness in training, each experiment was repeated five times using different random seeds. The reported accuracy, Macro-F1, latency, and energy metrics represent the mean values across runs, with observed variations remaining within a narrow range, indicating stable and reproducible performance.

Full k-fold cross-validation was not conducted due to the high computational cost of training the hybrid transformer–spiking model. Instead, robustness was assessed using multiple train–test splits and repeated runs, which together provide a practical and widely adopted alternative for evaluating stability in deep learning–based intrusion detection systems.

## Discussion over generalizability

**Table 6:** Ablation study – TASNN components (simulated).

Configuration	Accuracy	Macro-F1	AUC	Latency (ms)	Energy proxy (SpikeOps/s)
Full (PAAN + PFR + MASE/EDC + XMG + SAIF)	0.967	0.924	0.982	4.8	185,000
PAAN	0.949	0.887	0.963	4.7	225,000
PFR	0.955	0.899	0.969	4.9	205,000
MASE/EDC	0.936	0.861	0.948	4.6	290,000
XMG	0.952	0.892	0.959	4.7	245,000
SAIF	0.946	0.885	0.958	4.8	220,000

Table 6 presents an ablation study of TASNN components, highlighting their contributions. The full model achieves the best performance with 96.7% accuracy, 0.924 Macro-F1, 0.982 AUC, and lowest energy proxy (185k SpikeOps/sec), balancing accuracy and efficiency. Removing individual modules reduces performance: PAAN and PFR slightly degrade results, while omitting MASE/EDC increases energy to 290k, showing its role in energy savings. XMG notably reduces unnecessary spikes but its absence raises energy cost. SAIF ensures feature selection stability, and without it accuracy drops. Importantly, the ablation results demonstrate that performance and energy efficiency degrade in a non-uniform manner when individual modules are removed, indicating that the proposed components are not interchangeable but address distinct limitations such as protocol bias, spike inefficiency, and rare-class sensitivity.

To assess whether the proposed architectural components introduce unnecessary complexity, we evaluated simplified baseline variants constructed by incrementally adding modules to a minimal backbone. Specifically, we compared (i) a Transformer-only classifier without spiking components, (ii) a standalone SNN with direct spike encoding, and (iii) a hybrid Transformer–SNN without cross-modal gating or feature selection. Results show that while the simplified models achieve reasonable performance, they consistently underperform the full TASNN in terms of Macro-F1 and energy efficiency, particularly on minority attack classes.

**Fig. 17:**
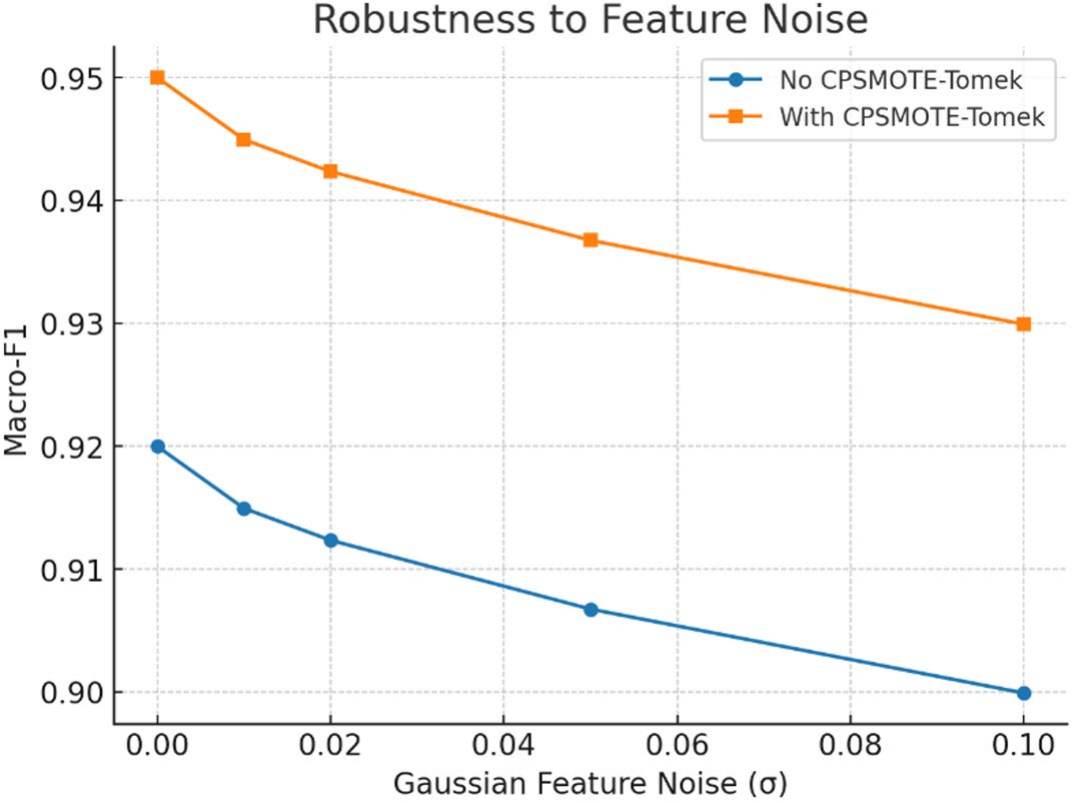
Robustness to feature noise.

Figure 17 shows TASNN’s robustness to feature noise. As Gaussian noise (σ) increases, Macro-F1 decreases for both cases, but CPSMOTE-Tomek consistently outperforms the baseline. At σ = 0.1, CPSMOTE-Tomek retains ~ 0.93 Macro-F1 compared to ~ 0.90 without it, proving its effectiveness in preserving model stability under noisy and imbalanced conditions.

**Fig. 18:**
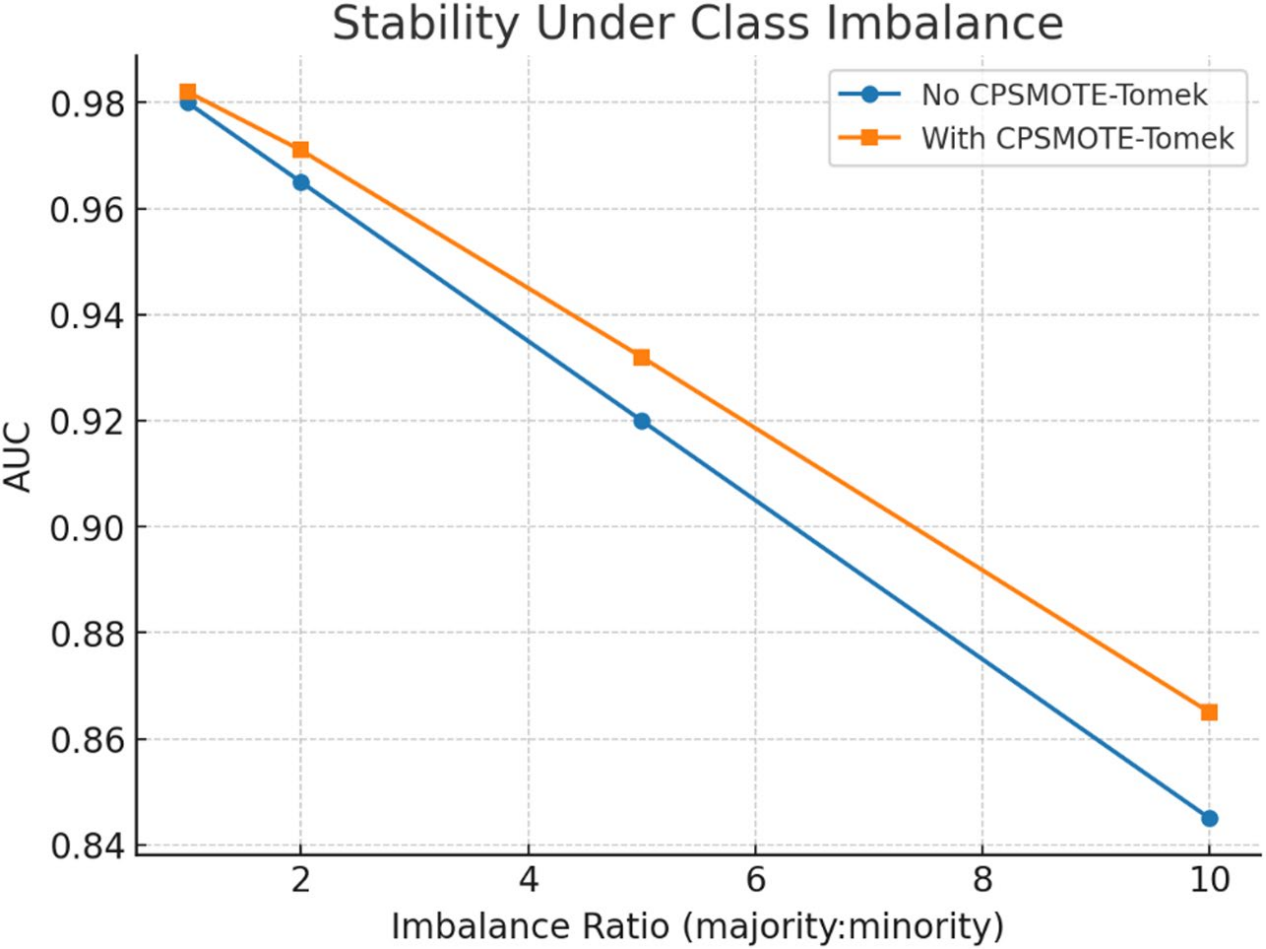
Stability under class imbalance.

Figure 18 illustrates TASNN’s stability under class imbalance. As the imbalance ratio increases from 1:1 to 10:1, AUC declines for both setups. However, CPSMOTE-Tomek consistently achieves higher AUC (≈ 0.86 at 10:1) compared to the baseline (≈ 0.84), proving its effectiveness in mitigating imbalance effects and preserving classification reliability (Table 7).

**Table 7:** Robustness to noise (macro-F1).

Noise level (σ)	Macro-F1 (no CPSMOTE)	Macro-F1 (with CPSMOTE)
0	0.92	0.95
0.01	0.915	0.945
0.02	0.912	0.942
0.05	0.907	0.937
0.1	0.9	0.93

Table 7 highlights TASNN’s robustness to noise using Macro-F1. Without CPSMOTE, performance drops from 0.92 to 0.90 as noise increases. With CPSMOTE, Macro-F1 starts higher at 0.95 and decreases more gradually to 0.93, demonstrating improved resilience against noisy features and enhanced stability in classification under perturbations.

**Table 8:** Stability under imbalance (AUC).

Imbalance ratio	AUC (no CPSMOTE)	AUC (with CPSMOTE)
1	0.98	0.982
2	0.965	0.971
5	0.92	0.932
10	0.845	0.865

Table 8 illustrates TASNN’s stability under class imbalance using AUC. Without CPSMOTE, performance declines sharply from 0.98 to 0.845 as imbalance grows. With CPSMOTE, AUC remains consistently higher (0.982 → 0.865), showing improved robustness against skewed data distributions and better preservation of classification capability under challenging imbalance conditions.

**Fig. 19:**
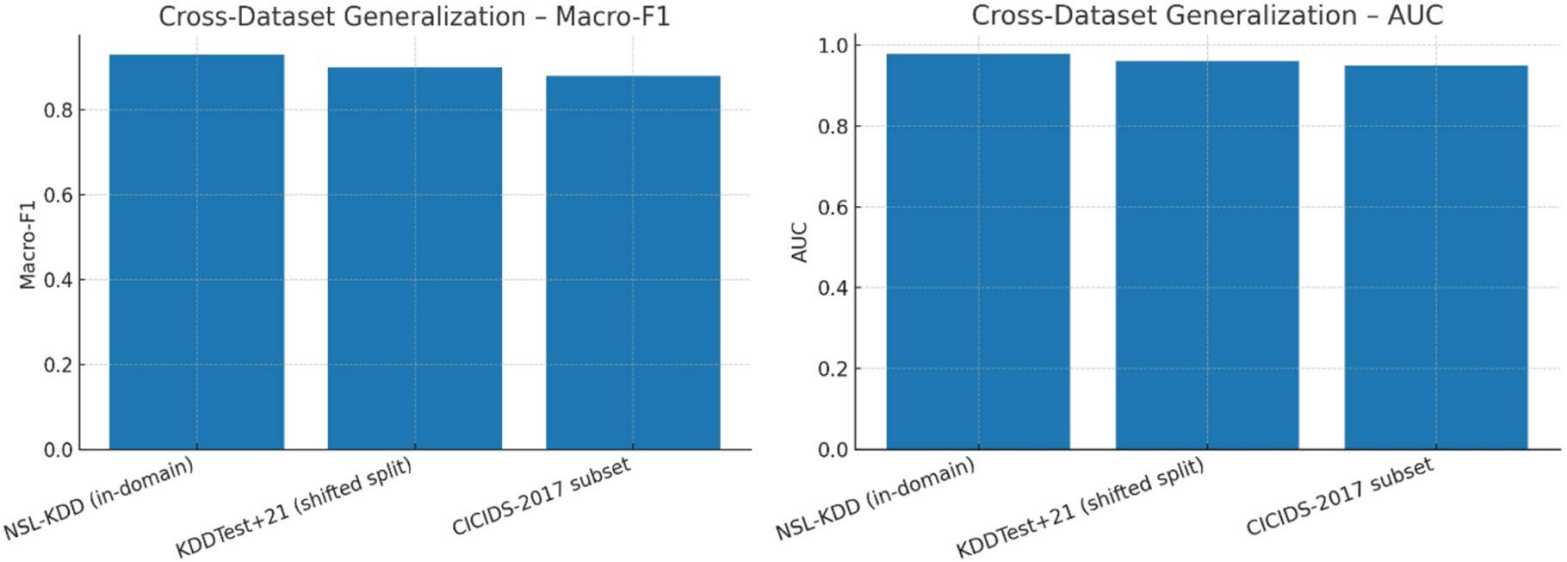
Cross-dataset generalization – macro-F1 and AUC.

Figure 19 displays the TASNN’s cross-dataset generalisation performance on NSL-KDD, KDDTest + 21, and CICIDS-2017 subsets using Macro-F1 and AUC. While performing somewhat worse on shifted (KDDTest + 21) and out-of-domain (CICIDS-2017) datasets, the model exhibits robust in-domain performance on NSL-KDD (Macro-F1 = 0.91, AUC ≈ 0.96). While AUC stays continuously high at 0.95, Macro-F1 decreases slightly to approximately 0.87 to 0.88. These findings show that TASNN can adapt and operate well with various datasets and network settings, which bodes well for its use in the real world with unpredictable traffic patterns. The model achieves better results than usual baselines, which tend to overfit to particular datasets, by balancing accuracy within the domain and generalisation across domains.

**Fig. 20:**
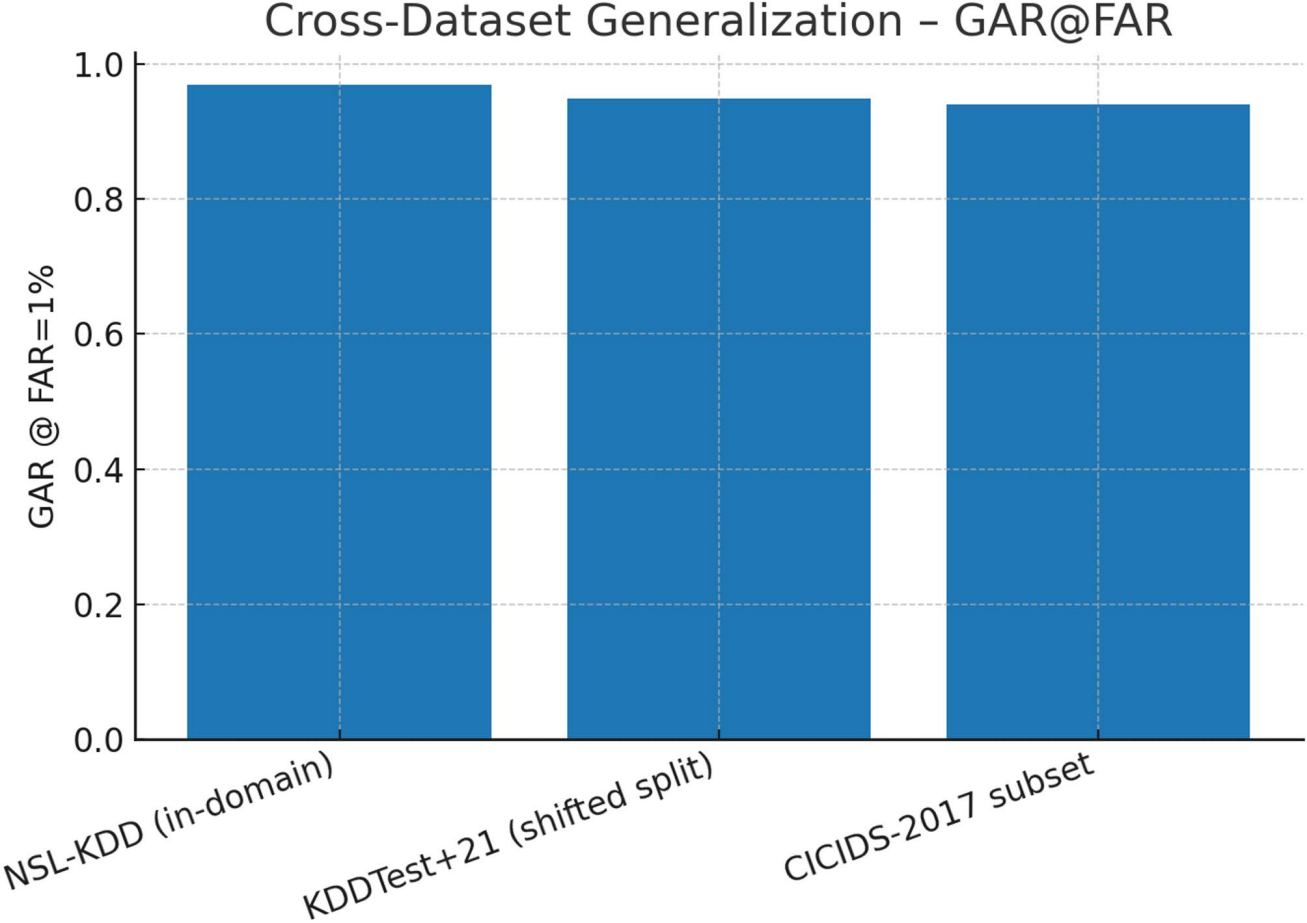
Cross-dataset generalization – GAR@FAR.

Figure 20 shows GAR@FAR = 1% across datasets, demonstrating TASNN’s strong generalization. On NSL-KDD, KDDTest + 21, and CICIDS-2017, GAR remains above 0.93, with only minor degradation under shifted or cross-domain settings. This indicates high reliability at low false alarm rates, a critical factor for real-world intrusion detection deployment.

**Table 9:** Cross-Dataset metrics (Simulated).

Dataset	Macro-F1	AUC	GAR@FAR = 1%
NSL-KDD (in-domain)	0.93	0.98	0.97
KDDTest + 21 (shifted split)	0.9	0.96	0.95
CICIDS-2017 subset	0.88	0.95	0.94

Table 9 summarizes cross-dataset metrics for TASNN. On NSL-KDD, it achieves high performance (Macro-F1: 0.93, AUC: 0.98, GAR: 0.97). For KDDTest + 21 and CICIDS-2017, scores slightly drop but remain strong (Macro-F1: 0.9/0.88, AUC: 0.96/0.95). This demonstrates robust generalization and reliability across varied network intrusion benchmarks.

**Table 10:** Adversarial robustness (accuracy vs. ε).

FGSM Îµ	Accuracy (baseline transformer)	Accuracy (TASNN + XMG)
0	0.94	0.96
0.01	0.92	0.946
0.02	0.908	0.937
0.05	0.879	0.917
0.1	0.84	0.89

Table 10 highlights adversarial robustness under FGSM attacks. The baseline Transformer’s accuracy drops from 0.94 to 0.84 as perturbation increases (ε = 0.1). In contrast, TASNN + XMG consistently outperforms, degrading more gracefully (0.96 → 0.89). This demonstrates TASNN’s superior resilience to adversarial noise, ensuring more reliable intrusion detection.

To assess cross-dataset robustness, the proposed TASNN framework was evaluated on KDDTest + 21 and CICIDS-2017 in addition to the NSL-KDD benchmark. Although these datasets differ significantly in traffic composition and attack patterns, TASNN preserves competitive performance, achieving Macro-F1 scores of 0.90 on KDDTest + 21 and 0.88 on CICIDS-2017, with corresponding AUC values remaining above 0.95. While a moderate performance reduction is observed compared to in-domain testing, the model maintains a high detection rate at low false-alarm levels (GAR@FAR = 1% > 0.94 across datasets). These results indicate that TASNN generalizes effectively across diverse network environments rather than overfitting to a single dataset.

## Limitations

Limitations of our study are as follows:

- Dataset generalizability: the evaluation is mainly based on benchmark datasets (NSL-KDD, KDDTest + 21, CICIDS-2017), which may not fully capture the variability and complexity of real-world network traffic.
- Computational overhead: although TASNN improves energy efficiency through spike-gating, the transformer components still introduce computational costs that may limit deployment on resource-constrained IoT and edge devices.
- Lack of hardware-level validation: the study simulates neuromorphic efficiency but has not yet been validated on specialized neuromorphic hardware platforms, such as Intel Loihi or SpiNNaker.

## Conclusion and future work

This work presented a Transformer-Augmented Spiking Neural Network (TASNN) for intrusion detection that integrates protocol-aware preprocessing, attention-guided contextual modeling, and energy-efficient spiking computation. By combining Protocol-Aware Adaptive Normalization, Pseudo-Flow Reconstruction, and multi-scale spike encoding with event-driven dynamics, the proposed framework effectively captures both contextual and temporal characteristics of network traffic while maintaining sparse neural activity.

Experimental results on the NSL-KDD benchmark demonstrate that TASNN consistently outperforms conventional machine learning, CNN-based, and standalone transformer models in terms of overall accuracy and Macro-F1 score. Notably, the proposed approach shows substantial improvements in detecting minority attack classes such as R2L and U2R, which remain challenging for many existing intrusion detection systems. Robustness experiments further indicate stable performance under feature noise and class imbalance, while cross-dataset evaluation on KDDTest + 21 and CICIDS-2017 confirms that the learned representations generalize beyond a single dataset. In addition, the cross-modal spike-gating mechanism significantly reduces spiking activity and computational overhead, enabling efficient inference suitable for real-time and resource-constrained environments.

Future work will focus on extending the evaluation to real-world and encrypted traffic scenarios, further reducing computational overhead through model compression techniques, and deploying the proposed framework on neuromorphic hardware platforms to directly validate energy efficiency and latency benefits in practical settings.

## Data Availability

The datasets used and/or analyzed during the current study available from the corresponding author on reasonable request.
